# Activation of the receptor KIT induces the secretion of exosome‐like small extracellular vesicles

**DOI:** 10.1002/jex2.139

**Published:** 2024-01-23

**Authors:** Annika Pfeiffer, Geethani Bandara, Jennifer D. Petersen, Guido H. Falduto, Joshua Zimmerberg, Dean D. Metcalfe, Ana Olivera

**Affiliations:** ^1^ Mast Cell Biology Section Laboratory of Allergic Diseases National Institute of Allergy and Infectious Diseases National Institutes of Health Bethesda Maryland USA; ^2^ Section on Integrative Biophysics Division of Basic and Translational Biophysics Eunice Kennedy Shriver National Institute of Child Health and Human Development National Institutes of Health Bethesda Maryland USA; ^3^ Inherited Movement Disorders Unit Neurogenetics Branch National Institute of Neurological Disorders and Stroke National Institutes of Health Bethesda Maryland USA

**Keywords:** cell activation, exosome, extracellular vesicle, KIT, mast cell, signalling pathway, stem cell factor

## Abstract

The receptor tyrosine kinase (RTK) KIT and its ligand stem cell factor (SCF) are essential for human mast cell (huMC) survival and proliferation. HuMCs expressing oncogenic KIT variants secrete large numbers of extracellular vesicles (EVs). The role KIT plays in regulating EV secretion has not been examined. Here, we investigated the effects of stimulation or inhibition of KIT activity on the secretion of small EVs (sEVs). In huMCs expressing constitutively active KIT, the quantity and quality of secreted sEVs positively correlated with the activity status of KIT. SCF‐mediated stimulation of KIT in huMCs or murine MCs, or of transiently expressed KIT in HeLa cells, enhanced the release of sEVs expressing exosome markers. In contrast, ligand‐mediated stimulation of the RTK EGFR in HeLa cells did not affect sEV secretion. The release of sEVs induced by either constitutively active or ligand‐activated KIT was remarkably decreased when cells were treated with KIT inhibitors, concomitant with reduced exosome markers in sEVs. Similarly, inhibition of oncogenic KIT signalling kinases like PI3K, and MAPK significantly reduced the secretion of sEVs. Thus, activation of KIT and its early signalling cascades stimulate the secretion of exosome‐like sEVs in a regulated fashion, which may have implications for KIT‐driven functions.

## 1 INTRODUCTION

Extracellular vesicles (EVs) are cell‐secreted nanovesicles loaded with selected molecular cargo such as lipids, nucleic acids and proteins that may reflect the status of the cell of origin. After their secretion, EVs may transfer donor cell cargo to recipient cells and potentially modify functional responses. As such, EVs are intercellular communicators conveying messages to proximal or distal tissue environments. While there is a vast heterogeneity regarding their biogenesis, size and composition, the most studied types of EVs are ectosomes (often also referred to as microvesicles) and exosomes. Ectosomes directly bud from the plasma membrane and, although there are no definite consensus protein markers for EV subtypes yet, annexin A1, ARF6 and basigin are considered characteristic of ectosomes (Clancy et al., 2019; Jeppesen et al., 2019; Mathieu et al., 2021; Muralidharan‐Chari et al., 2009). Exosomes originate from the endosomal compartment by the initial formation of intraluminal vesicles (ILVs) from endosome membranes, which are released as exosomes when multivesicular bodies (MVBs) fuse with the plasma membrane. EV protein markers such as CD63 and syntenin‐1 have persistently been used to define and study exosomes (Kowal et al., 2016; Kugeratski et al., 2021; Mathieu et al., 2021).

The quantity and composition of secreted EVs are dynamic and can be modulated in response to cell‐activating stimuli (Groot Kormelink et al., 2016; Nolte‐’t Hoen et al., 2013; Segura et al., 2005; van der Vlist et al., 2012). Activation of immune cell receptors such as the T cell receptor (TCR), B cell receptor (BCR), toll‐like receptor (TLR) on dendritic cells, or FcεRI, the high affinity IgE receptor expressed on mast cells (MCs), triggers the release of EV populations that differ in quantity and/or molecular content from vesicles secreted by unstimulated cells (Blanchard et al., 2002; Groot Kormelink et al., 2016; Nolte‐’t Hoen et al., 2013; Rialland et al., 2006; Segura et al., 2005; van der Vlist et al., 2012).

MCs are innate immune cells that contribute to allergic inflammation, especially through the release of soluble pro‐inflammatory mediators stored in MC granules after activation of FcεRI by IgE/antigen complexes. Both resting and activated MCs also secrete EVs (Groot Kormelink et al., 2016; Liang et al., 2020; Molfetta et al., 2020; Raposo et al., 1997; Skokos et al., 2001). MCs express a plethora of receptors that regulate various MC responses (Falduto et al., 2021), and while activation of the receptor MRGPRX2 triggers enhanced secretion of EVs, LPS‐mediated stimulation of TLR4 does not affect the quantity of secreted EVs, suggesting that a triggered change in EV release is dependent on the MC stimulus (Groot Kormelink et al., 2016). Stem cell factor (SCF), the ligand for the receptor tyrosine kinase (RTK) KIT, is essential for the differentiation, survival and proliferation of MCs and does not by itself at physiological levels induce degranulation in human MCs (huMCs) (Hundley et al., 2004). KIT variants harbouring mutations that keep the receptor constitutively active have been associated with neoplastic MCs and MC disorders such as mastocytosis. Gastrointestinal stromal tumour (GIST) cells and neoplastic MCs expressing constitutively active KIT variants release abundant populations of EVs and disseminate KIT in both microvesicle‐ and exosome‐like EVs of distinct composition (Atay et al., 2018; Kim et al., 2018; Pfeiffer et al., 2022; Xiao et al., 2014), suggesting a potential role for active KIT in the regulation of EV secretion.

Here, we studied the effects of KIT activation on the quantity and characteristics of secreted small EVs (sEVs), which were isolated by differential ultracentrifugation. We demonstrate that activation of KIT in huMCs by SCF or gain‐of‐function mutations induces the secretion of more and smaller EVs that are enriched for proteins characteristic of exosomes. The stimulated release of sEVs was prevented by KIT inhibitors or inhibition of specific signalling pathways downstream of KIT, implicating early signalling events in the regulated secretion of sEVs. The enhanced SCF‐mediated sEV release was also observed in HeLa cells after transient expression of KIT, but not after stimulation of epidermal growth factor receptor (EGFR), an endogenously expressed RTK in HeLa cells. These data suggest that the KIT‐induced release of sEVs is not restricted to MCs and that KIT‐specific signalling components contributing to the release of sEVs may not be common to all RTKs. Our results support that the quantity and characteristics of secreted sEVs, and potentially their biological functions, can be dynamic and modulated by intracellular signalling in response to specific stimuli in the cellular microenvironment. Furthermore, the high secretion of sEVs from cells expressing oncogenic KIT is partly KIT‐signalling dependent and differs from constitutive EV secretion, which may have implications in the treatment of KIT‐driven diseases such as mastocytosis and GIST in which EVs are thought to contribute to disease pathology.

## 2 MATERIALS AND METHODS

### 2.1 Cell culture

Cells were maintained in a humidified incubator at 37°C and 5% CO_2_. The LAD2 huMC line, expressing normal KIT, was maintained in serum‐free StemPro‐34 media supplemented with StemPro‐34 nutrient supplement (Gibco), 2 mM L‐Glutamine, 100 IU/mL penicillin, 100 μg/mL streptomycin (Corning) and 100 ng/mL recombinant human SCF (rhSCF; R&D Systems) (Kirshenbaum et al., 2003). The neoplastic huMC lines HMC‐1.1 (KIT V560G) and HMC‐1.2 (KIT V560G, KIT D816V) were kindly provided by Dr. Butterfield (Mayo Clinic, Rochester, MN, USA) (Butterfield et al., 1988; Sundström et al., 2003). HMC‐1 cells were maintained in Iscove's modified Dulbecco's medium (IMDM, Corning) supplemented with 2 mM L‐Glutamine, 100 IU/mL penicillin, 100 μg/mL streptomycin (Corning) and 10% (v/v) fetal bovine serum (FBS, Gibco, Thermo Fisher Scientific). The MCBS cell lines originated from an immortalised non‐KIT expressing murine bone marrow mast cell line that was stably transfected with empty vector or human wild‐type KIT and cultured as described (Smrž et al., 2013). Adherent HeLa cells (ATCC) were cultured in DMEM (Corning) containing 10% (v/v) FBS, 100 IU/mL penicillin, and 100 μg/mL streptomycin (Corning), and passaged by trypsinization (Corning). Cell counts, and viability (acridine orange/propidium iodide stain, Logos Biosystems) were assessed on a Luna‐FL automated brightfield and dual fluorescence cell counter (Logos Biosystems).

### 2.2 Inhibitors

The KIT inhibitors imatinib, dasatinib and midostaurin, the EGFR inhibitor gefitinib, and the JAK2 inhibitor fedratinib were used at 1 μM (Selleck Chemicals). The PI3K inhibitor LY294002 and the MAPK inhibitor U0126 were used at 10 μM (Selleck Chemicals). The dynamin inhibitor dynasore was used at 100 μM and the clathrin inhibitor Pitstop2 was used at 30 μM (Sigma‐Aldrich).

### 2.3 Cell transfection with plasmid DNA

HeLa cells were transiently transfected in a 150 mm dish with pcDNA3.1‐KIT or the corresponding empty vector (Chan et al., 2013) using Lipofectamine 3000 (Thermo Fisher Scientific) according to the manufacturer's instructions and used for EV secretion assays at 24 h post‐transfection.

### 2.4 Secretion of mast cell EVs

For the collection of MC‐EVs, LAD2 cells were cultured overnight in cytokine‐free StemPro‐34 media (with nutrient supplement). The following day, LAD2 cells were washed with HEPES buffer and 10 to 15 × 10^6^ cells were incubated for 2 h at 37°C in 13 mL HEPES‐BSA buffer (10 mM HEPES, 137 mM NaCl, 2.7 mM KCl, 0.4 mM Na_2_HPO_4_·7 H_2_O, 5.6 mM glucose, 1.8 mM CaCl_2_·2H_2_O, 1.3 mM MgSO_4_·7H_2_O and 0.04% BSA [pH 7.4]) with or without rhSCF (100 ng/mL). Where indicated, LAD2 cells were primed with 100 ng/mL biotinylated IgE overnight in regular SCF‐containing media, washed with HEPES buffer, and activated by 100 ng/mL streptavidin (antigen) the next day. HEPES‐BSA buffer, commonly used for MC assays (Kuehn et al., 2010), was pre‐filtered through a 0.22 μm PES membrane filter and used instead of cell media to avoid co‐isolation of residual EVs or protein from the culture media. 10 to 15 × 10^6^ MCBS, HMC‐1.1 or HMC‐1.2 cells were likewise transferred to HEPES‐BSA buffer and allowed to secrete EVs for 2 h in the absence or presence of rhSCF as indicated. Where specified, cells were pre‐incubated with inhibitors for 30 min in regular media, followed by incubation in inhibitor‐supplemented HEPES‐BSA buffer. Supernatants and cells were collected after 2 h and EVs released into the supernatant were isolated by differential ultracentrifugation as described below.

### 2.5 Secretion of HeLa EVs

For the collection of EVs, the growth media of 15 × 10^6^ HeLa cells plated in 150 mm cell culture dishes was replaced with DMEM containing EV‐depleted FBS (obtained by ultracentrifugation at 120,000 × *g* for 24 h; SW32‐Ti rotor, OptimaXE‐90, Beckman Coulter). HeLa cells were incubated for 2 h with 100 ng/mL EGF (R&D Systems) to activate EGFR or with 100 ng/mL rhSCF to stimulate transiently expressed KIT. Supernatants were collected after 2 h and EVs secreted into the media were isolated by differential ultracentrifugation as described below.

### 2.6 Isolation of EVs by differential ultracentrifugation

All steps were carried out at 4°C or on ice and included cold filtered 1x PBS obtained by using a 0.22 μm PES membrane filter. EVs were isolated as previously described (Pfeiffer et al., 2022). In brief, cell supernatant containing secreted EVs was sequentially processed. Cells were first pelleted at 400 × *g* for 10 min before the supernatant was sedimented at 2000 × *g* for 20 min to remove debris and apoptotic bodies. Large EVs (lEVs) were pelleted at 15,000 × *g* for 40 min (Sorvall Legend XTR, Fiberlite F15‐8 × 50cy rotor, fixed angle) followed by sedimentation of the supernatant at 120,000 × *g* for 2 h to obtain small EVs (sEVs) using an SW40‐Ti rotor (OptimaXE‐90, Beckman Coulter). Pelleted EVs were resuspended in 20 μL PBS and used immediately for analyses.

### 2.7 Density gradients

sEVs obtained from HMC‐1.1 or HMC‐1.2 cells were separated by iodixanol density gradients as described (Pfeiffer et al., 2022). EVs pelleted from each of the ten fractions were re‐suspended in RIPA buffer (Cell Signaling Technology), combined with 4 × LDS sample buffer (Invitrogen, Thermo Fisher Scientific), and analysed by immunoblotting as described below.

### 2.8 Nanoparticle tracking analysis (NTA)

All EV preparations were analysed by NTA to obtain particle concentrations and size distributions. Measurements were performed on a NanoSight NS300 instrument (Malvern Panalytical). Diluted EV samples (1:500 in filtered PBS) were injected into the laser chamber at a constant infusion rate by a syringe pump (Harvard Apparatus). Particles were captured at room temperature for 30 s with five repetitions with a camera level of 11–12. NTA software (NTA 3.3 Dev Build 3.3.301) was used for recording, data processing and calculation of the concentration (particles/mL) and particle diameter (nm) for each repetition and the average of five dynamic measurements. The concentration of the EV samples was corrected by the dilution factor. For plotting, secreted EVs were calculated per 10^6^ cells. Each data point in the graphs represents the average from five repetitive NTA measurements from a single experiment. The EV size is represented by the average mode (highest peak).

### 2.9 Measurement of mast cell degranulation by monitoring β‐hexosaminidase release

MC degranulation was determined by β‐hexosaminidase released into the cell supernatant (Kuehn et al., 2010). LAD2 cells were incubated overnight in cytokine‐free media with or without 100 ng/mL biotinylated human myeloma IgE (Millipore, MA; biotinylated in‐house). The following day, cells were rinsed twice with HEPES‐BSA buffer and stimulated with rhSCF (100 ng/mL), streptavidin (antigen; 100 ng/mL), or a combination of both for 2 h at 37°C. β‐Hexosaminidase release was calculated as the percentage of the total cellular β‐hexosaminidase content (Kuehn et al., 2010).

### 2.10 Immunoblotting

Cell or EV lysates were prepared on ice in RIPA buffer (Cell Signaling Technology) supplemented with Halt EDTA‐free protease and phosphatase inhibitor cocktail (Thermo Fisher Scientific) for 10 min with occasional vortexing. Detergent‐insoluble material was pelleted at 13,000 rpm for 10 min at 4°C. The protein concentration of lysates was determined with a bicinchoninic acid (BCA) protein assay (Pierce, Thermo Scientific) or using the Qubit protein assay kit (Thermo Fisher Scientific). Cleared lysates were combined with appropriate volumes of 4x LDS sample buffer (Invitrogen, Thermo Fisher Scientific), sonicated in a water bath sonicator for 30 s, and heated at 40°C for 10 min. Equal amounts of protein were loaded and separated on NuPAGE Bis‐Tris 4%–12% gels (Thermo Fisher Scientific). Transfer to nitrocellulose membranes was performed in a turbo transfer system (Biorad) at 25 V for 10 min. Membranes were air‐dried for 10 min, followed by blocking for 1 h in Odyssey blocking buffer (formulated in TBS, LI‐COR Biosciences). Membranes were incubated in primary antibody dilutions made in blocking buffer overnight at 4°C on a shaker. The following primary antibodies were used at a dilution of 1:1000 unless stated otherwise: goat anti‐KIT (R&D Systems, AF332), rabbit anti‐pKIT Tyr823 (Cell Signaling Technologies, 77522), mouse anti‐JAK2 (Invitrogen, MA5‐15632), rabbit anti‐pJAK2 Tyr1007/1008 (Cell Signaling Technologies, 3771), mouse anti‐AKT (Cell Signaling Technologies, 2920), rabbit anti‐pAKT Ser473 (Cell Signaling Technologies, 9271), mouse anti‐ERK (Cell Signaling Technologies, 4696), rabbit anti‐pERK Thr202/Tyr204 (Cell Signaling Technologies, 9101), rabbit anti‐EGFR (Cell Signaling Technologies, 4267), mouse anti‐pEGFR Tyr1068 (Cell Signaling Technologies, 2236), rabbit anti‐Syntenin‐1 (Abcam, ab133267), mouse anti‐CD63 (BD Bioscience, 556019), rabbit anti‐ALIX (Abcam, ab186429), mouse anti‐CD81 (R&D Systems, MAB4615), mouse anti‐Basigin (Thermo Fisher Scientific, 66443‐1‐IG), rabbit anti‐Annexin A1 (Abcam, ab214486; 1:2000), rabbit anti‐ARF6 (Abcam, ab226389), mouse anti‐β‐actin (Sigma‐Aldrich, A5441; 1:10,000). Washing was performed three times with TBS‐T (TBS with 0.1% Tween‐20) for 5 min each. Secondary antibodies were donkey anti‐mouse IRDye680RD or IRDye800CW, donkey anti‐goat IRDye680RD or IRDye800CW, donkey anti‐rabbit IRDye680RD or IRDye800CW (LI‐COR Biosciences), used at a dilution of 1:20,000 in TBS‐T/blocking buffer for 1 h at room temperature. Antibody‐probed membranes were washed three times with TBS‐T, rinsed with TBS and imaged with a LI‐COR Odyssey CLx scanner. Analysis and quantification were performed using the Image Studio Lite software (version 5.2, Li‐Cor). Shown immunoblots are representative of at least three independent experiments.

### 2.11 Electron microscopy (EM)

Reagents for the preparation of HMC‐1 MCs for thin section EM were obtained from Electron Microscopy Sciences, and all steps were carried out at room temperature unless stated otherwise. Cells were plated on plastic coverslips prepared by cutting a sheet of Aclar 33C film into rectangular coverslips to fit into 12‐well plates and were rinsed with distilled water before sterilised in 70% ethanol. Coverslips were rinsed ten times with cell culture grade sterile water (Thermo Fisher Scientific) and coated with poly‐l‐lysine (Sigma‐Aldrich) at 4°C. HMC‐1.1 and HMC‐1.2 cells were plated on coverslips and after 24 h, cells were fixed with 2% paraformaldehyde and 2% glutaraldehyde in 0.1 M sodium cacodylate buffer containing 2 mM calcium chloride for 2 h. Next, cells were rinsed with cacodylate buffer and postfixed for 40 min with 0.25% osmium and 0.25% potassium ferrocyanide (Fisher Scientific) in cacodylate buffer. After rinsing with cacodylate buffer, cells were incubated in 0.5% tannic acid in cacodylate buffer for 30 min, rinsed with 50 mM acetate buffer at pH 5.2, and then stained with 2% uranyl acetate in acetate buffer for 30 min. Cells were rinsed with acetate buffer and then dehydrated with a graded series of 5 min ethanol rinses (50%, 75%, 95%, and twice with 100% ethanol). Cells were infiltrated with EmBED812 resin mixed with ethanol (50%, 75%, 95%, and twice with 100% resin). Each infiltration step lasted for at least 1 h. To embed coverslips, gelatin capsules (size 0) with the caps removed, were filled with resin, inverted and placed over the cells on the coverslip, and then polymerised in a 60°C oven for 24–48 h. After polymerisation, the gelatin capsules were separated with a jewellers saw into individual blocks. The area around embedded cells in each block were trimmed with a razor blade, and the Aclar coverslip was peeled off the resin, exposing embedded cells on the surface of the resin block. Sections of embedded cells were cut en face with an ultramicrotome (EM UC7, Leica Microsystems, Wetzlar, Germany) to a thickness of 70 nm using a diamond knife (DiATOME, Hatfield, PA, USA) and picked up on 2 × 1 mm formvar and carbon‐coated slot grids. Sections were viewed using a JEOL1400 transmission electron microscope (JEOL USA, Peabody, MA USA) operated at 120 KeV. Images were collected with a BioSprint 29 CMOS TEM Camera (AMT Imaging, Woburn, MA, USA).

Images were collected and analysed under blind conditions. To collect images of the two cell lines for analysis, a section of each cell line was scanned at a low magnification (500×) and positions of 25–30 cells per cell line were saved on the microscope. Each cell was then imaged at direct magnification of 2000 or 2500× to capture the cell in its entirety for area measurements. Then, the cell was imaged at a direct magnification of 6000× to provide views of the cytoplasmic organelles, such as MVBs.

Fiji image analysis software (Schindelin et al., 2012), a version of ImageJ (Rueden et al., 2017), was used to calculate the cell areas and quantify MVB‐like organelles per area of cytoplasm per cell. To calculate the area of cytoplasm per cell, a region was drawn around the cell periphery and the area occupied by the nucleus, if present, was also traced and subtracted from the total cell area to determine the area of cytoplasm. To count the number of MVB‐like organelles present per cell, the higher magnification images were used to trace regions around organelles in the cytoplasm that met the following characteristics: (1) were roughly spherical, (2) had electron lucent lumen, (3) contained some or many vesicles of light grey to dark lumen electron density. For each cell, the number of MVB‐like organelles traced was divided by the area of cytoplasm to calculate the number of MVBs per square micron per cell.

### 2.12 Statistical analysis

All quantifications and statistical analyses using GraphPad Prism (9.3.1) were performed from at least three independent experiments. Unpaired Student's *t*‐tests were applied to determine significance; *, *p* < 0.05; **, *p* < 0.01; ***, *p* < 0.001; ****, *p* < 0.0001; ns, not significant.

### 2.13 EV‐TRACK

We have submitted relevant details of EV‐related experiments to the EV‐TRACK knowledgebase (EV‐TRACK ID: EV230604) (Van Deun et al., 2017).

## 3 RESULTS

### 3.1 Phosphorylation levels of oncogenic KIT variants in neoplastic HMC‐1 cells positively correlate with the secretion of more and smaller sEVs

**FIGURE 1:**
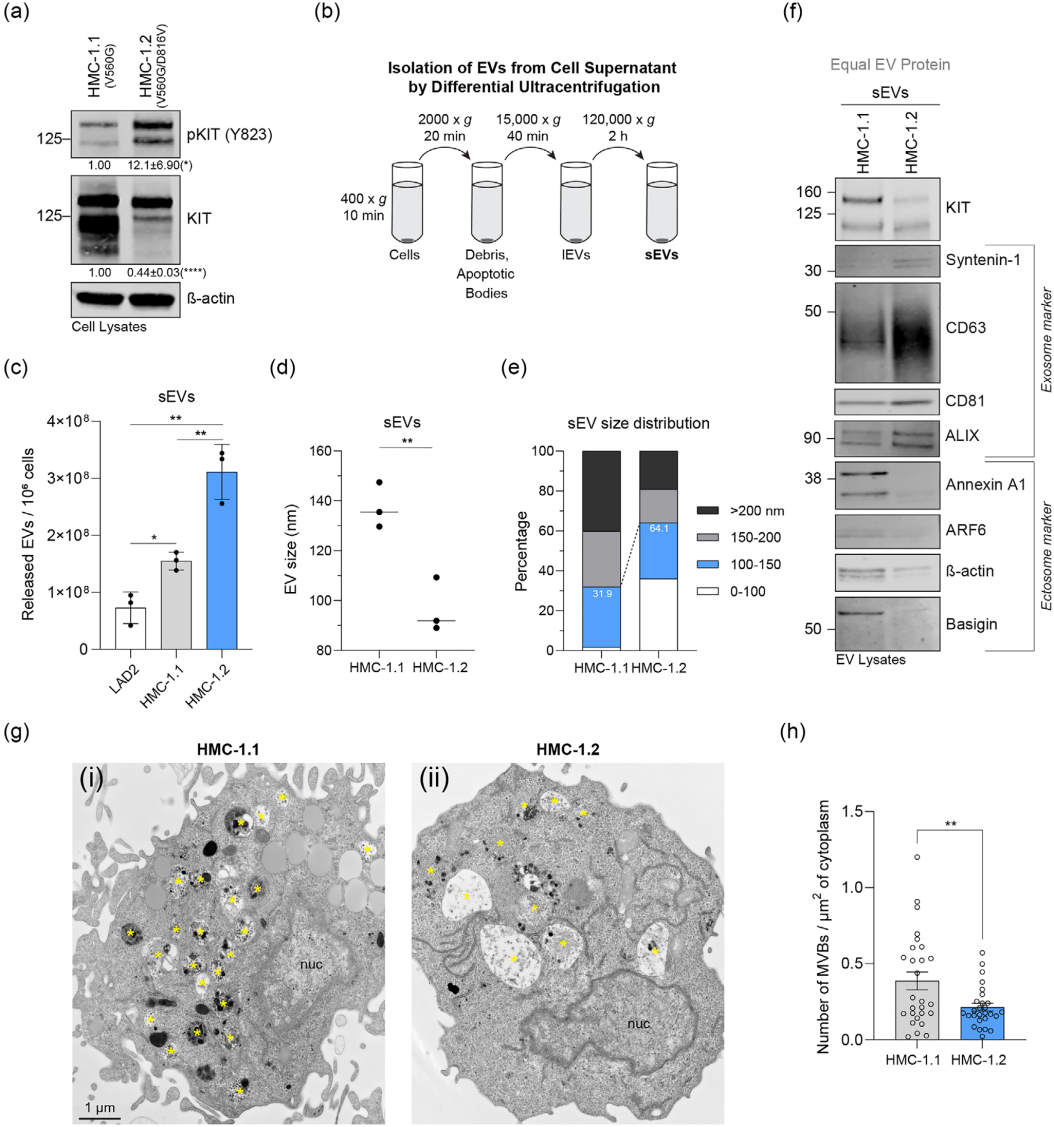
Enhanced KIT phosphorylation in MCs expressing oncogenic KIT variants coincides with the increased secretion of exosome‐like sEVs. (a) Cell lysates (30 μg of protein) of the HMC‐1.1 and HMC‐1.2 cell lines (carrying the V560G and V560G/D816V mutations in KIT, respectively) were analysed by Western blotting with the indicated antibodies. The relative expression of pKIT/KIT or KIT was corrected by β‐actin signals, normalised to HMC‐1.1 cells, and indicated below the immunoblots. Data are represented as the mean ± SD from three independent experiments. (b) Schematic of the differential ultracentrifugation approach to isolate sEVs from cell supernatant. (c) Quantitative comparison of sEV secretion from three different huMC models under regular culture conditions. NTA‐obtained data are represented as the mean ± SD from three independent experiments. (d) Size of sEVs secreted from HMC‐1.1 and HMC‐1.2 cells. The line represents the median from three independent experiments. (e) Size distribution in percentage of sEVs released from HMC‐1.1 and HMC‐1.2 cells. Shown data represent the mean from three independent experiments. (f) Immunoblot analysis of sEV lysates derived from HMC‐1.1 or HMC‐1.2 cells. Equal EV protein (20 μg) was loaded. The indicated antibodies were used for signal detection and grouped in typical exosome or ectosome representing marker as highlighted. (g) Representative thin sections of HMC‐1.1 (i) and HMC‐1.2 (ii) cells. Asterisks (*) indicate MVB‐like organelles. (h) Number of MVBs per μm^2^ of cytoplasm was assessed from EM thin sections obtained for HMC‐1.1 and HMC‐1.2 cells. Data are represented as the mean ± S.E.M., *n* = 27 cells per cell line. *, *p* < 0.05; **, *p* < 0.01; ****, *p* < 0.0001 (unpaired *t* test). MVB, multivesicular bodies; NTA, nanoparticle tracking analysis; Nuc, nucleus; sEV, small extracellular vesicle.

Transformed huMCs expressing constitutively active KIT variants secrete large quantities of EVs, but it is not known whether KIT activation plays a role in regulating this EV secretion. The neoplastic huMC lines HMC‐1.1 and HMC‐1.2 express KIT with different gain‐of‐function mutations that keep the receptor constitutively active (Sundström et al., 2003), but do not express functional FcεRI and do not show consistent capacity for degranulation (Nilsson et al., 1994; Xia et al., 2011). We confirmed that HMC‐1.2 cells harbouring two oncogenic KIT mutations express higher levels of active, phosphorylated KIT than HMC‐1.1 cells. Concomitantly, total KIT protein levels were reduced in HMC‐1.2 cells, consistent with induced degradation of activated receptors (Figure 1a). To quantitatively compare the levels of EVs secreted by huMC lines, we sedimented the cell supernatants by differential ultracentrifugation to obtain lEVs and sEVs (Figure 1b). Correlating with increased phosphorylation levels of KIT, HMC‐1.2 cells secreted significantly more lEVs and sEVs than HMC‐1.1 cells (Figure S1a and Figure 1c). Additionally, both cell lines released significantly more sEVs than LAD2 huMCs, which express normal KIT (Figure 1c).

The median size of HMC‐1.2 sEVs (∼90 nm) was significantly smaller compared to HMC‐1.1 sEVs (∼135 nm) and the percentage of sEVs smaller than 150 nm was doubled compared to HMC‐1.1 sEVs (Figure 1d,e). Immunoblot analyses revealed that exosome markers (CD63, ALIX and syntenin‐1) were highly expressed in HMC‐1.2‐derived sEVs, while HMC‐1.1‐derived sEVs had a greater representation of EV protein markers associated with ectosomes such as annexin A1, ARF6 and basigin (Figure 1f). Thus, an increased proportion of small sized exosome‐like EVs among the total sEVs secreted from HMC‐1.2 cells would explain the smaller median size of HMC‐1.2 sEVs compared to HMC‐1.1 sEVs. Similar trends were observed for lEVs, suggesting a general predominance of exosome‐characteristic proteins in EVs released from HMC‐1.2 cells (Figure S1b,c), and we continued hereafter to focus our study on the more refined sEV population. The differences in the expression of EV marker proteins were not observed in total cell lysates (Figure S2), indicating that the sEV protein content is not a mere reflection of the protein abundance in the cells. Additionally, we examined sEVs released from HMC‐1.1 or HMC‐1.2 cells by iodixanol density gradients (Figure S3). We observed a shift of exosome marker‐containing sEVs to slightly denser fractions and an overall higher expression of exosome proteins in HMC‐1.2 compared to HMC‐1.1 sEV gradients, supporting our finding that HMC‐1.2‐derived sEVs are shifted towards exosome‐like EVs (Figure S3b and Figure 1f). Of note, while most HMC‐1.1‐derived KIT‐containing sEVs floated into fraction 1 co‐occurring with the strongest signal of ectosome‐like markers (Figure S3a), HMC‐1.2‐secreted KIT‐containing sEVs were distributed over fractions 1–4 overlapping with both ectosome‐ and exosome‐like protein markers (Figure S3b). These results are consistent with the conclusion that the differences in size and composition of sEVs secreted from HMC‐1.1 or HMC‐1.2 cells also impact the floating density.

We also imaged thin sections of HMC‐1.1 and HMC‐1.2 cells by electron microscopy to gain insight into the abundance of exosome‐containing MVBs at steady‐state (Figure 1g). As noted by others (Zabeo et al., 2017), MVBs in both cell lines were heterogeneous (Figure 1g). The number of MVBs in HMC‐1.2 cells was significantly lower compared to HMC‐1.1 cells (Figure 1h). An increased fusion rate of MVBs with the plasma membrane would potentially explain the reduced presence of MVBs in HMC‐1.2 cells and the observed higher secretion of exosome marker expressing sEVs. Together, these data reveal that higher levels of constitutively active KIT in neoplastic HMC‐1 cells correlate with increased numbers and decreased average sizes of secreted sEVs and appear to influence the EV subtype.

### 3.2 Modification of KIT activity results in altered sEV secretion

**FIGURE 2:**
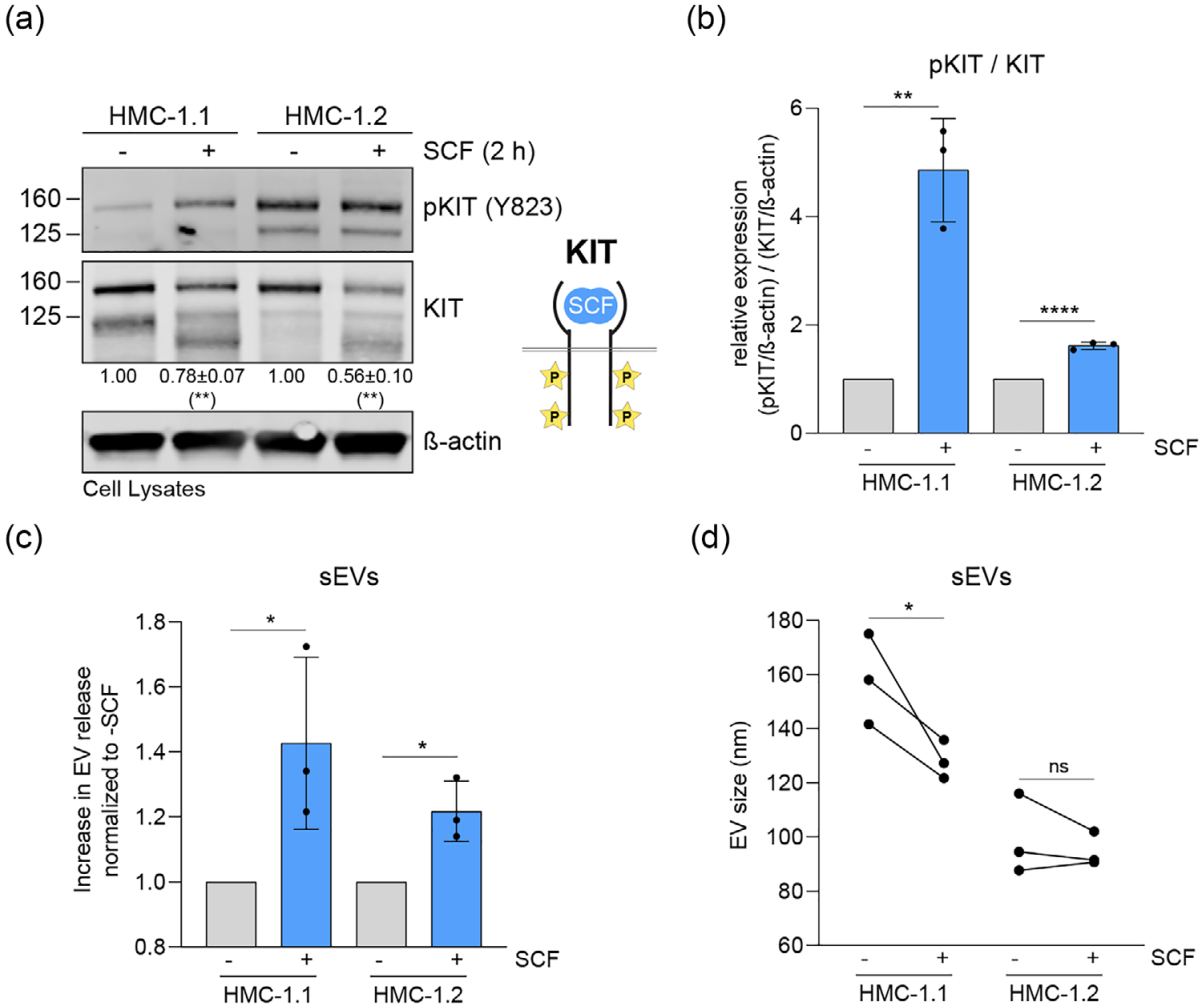
Additional SCF‐mediated stimulation of constitutively active KIT variants increases the secretion of sEVs. (a) Cell lysates (30 μg of protein) of HMC‐1.1 and HMC‐1.2 cells treated or not with SCF were analysed by immunoblotting with the indicated antibodies. The relative expression of KIT was corrected by β‐actin, normalised to unstimulated cells, and indicated below the immunoblot. Data are represented as the mean ± SD from three independent experiments. The schematic illustrates SCF‐binding to KIT leading to activation/ phosphorylation of the receptor. (b) Quantification of KIT phosphorylation (pKIT) in untreated or SCF‐stimulated HMC‐1.1 and HMC‐1.2 cells. The relative expression of pKIT/KIT was normalized to untreated cells in each cell line. Data are represented as the mean ± SD from three independent experiments. (c) Increase in sEV release from HMC‐1.1 or HMC‐1.2 cells after stimulation with SCF. Data are represented as the mean ± SD from three independent experiments. (d) Average size of sEVs secreted by untreated or SCF‐treated HMC‐1.1 or HMC‐1.2 cells. Data points from three independent experiments are plotted and connected by lines per independent experiment. *, *p* < 0.05; **, *p* < 0.01; ****, *p* < 0.0001 (unpaired *t* test). Ns, not significant; SCF, stem cell factor; sEV, small extracellular vesicle.

To substantiate the observation that the receptor activity of KIT is at least partially linked to the secretion of sEVs, we boosted mutant KIT activity by treating the HMC‐1 cell lines with the receptor ligand SCF and determined the quantity of released sEVs. SCF remarkably enhanced the phosphorylation of KIT in HMC‐1.1 cells, while in HMC‐1.2 cells the effect was subtle, probably because the baseline KIT phosphorylation is already near saturation (Figure 2a,b). Expectedly, the expression of total KIT decreased after SCF stimulation in both cell lines (Figure 2a), supporting induced receptor activation. Importantly, SCF treatment further increased the levels of released sEVs in both cell lines (Figure 2c). In addition, sEVs secreted from SCF‐stimulated HMC‐1.1 cells were significantly smaller than sEVs from untreated cells and closer in size to sEVs from HMC‐1.2 cells (Figure 2d), implying that boosting the activity of KIT increases the proportion of small sized sEVs among all secreted sEVs. HMC‐1.2‐derived sEVs did not significantly change in size after SCF treatment, which is consistent with a more saturated activation level of KIT (Figure 2d). These results strengthen the notion that the activation of the receptor KIT leads to a stimulated secretion of sEVs and a change in their physical properties.

**FIGURE 3:**
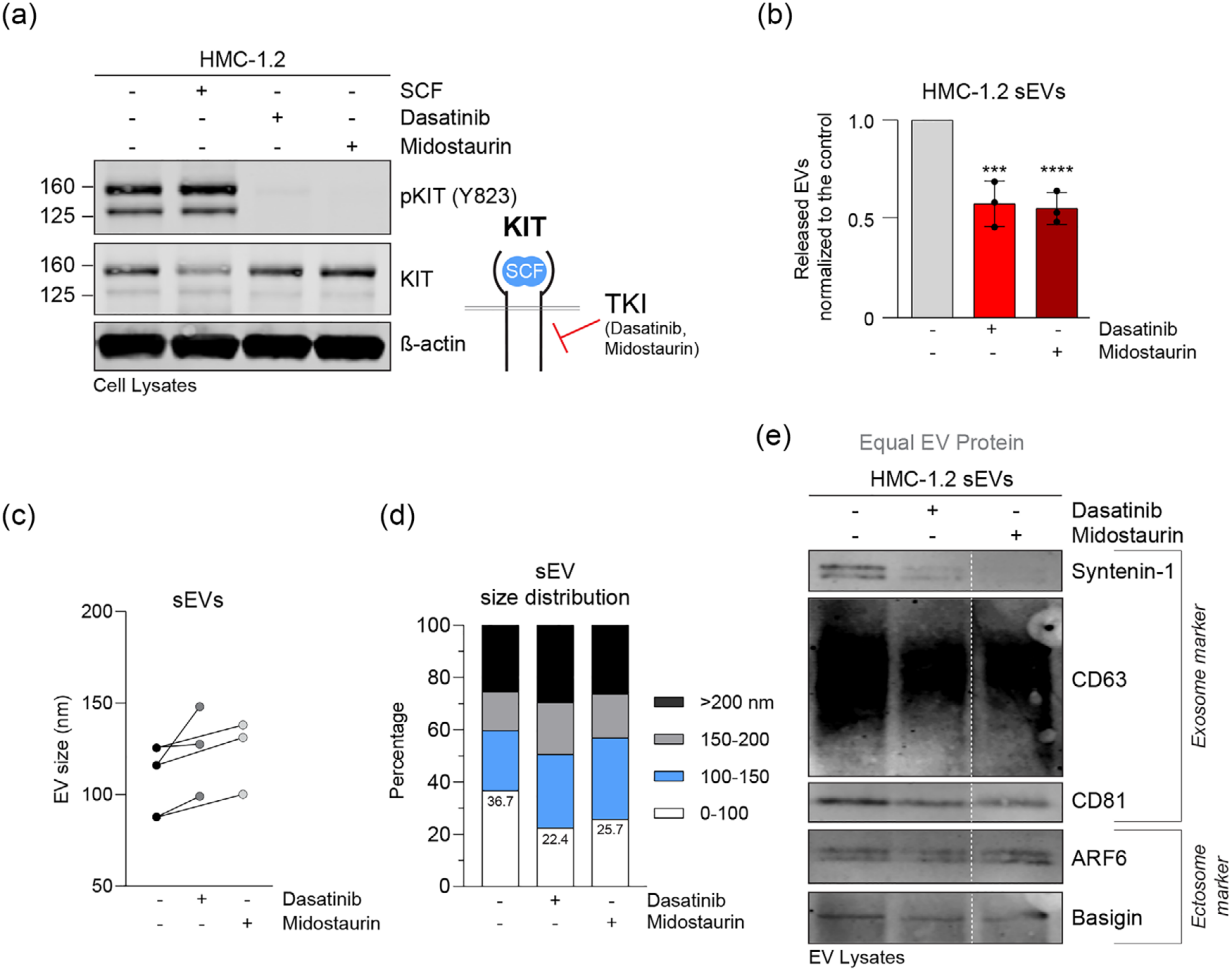
Inhibition of constitutive KIT activity decreases the quantity of released sEVs. (a) Lysates (30 μg of protein) of HMC‐1.2 cells treated for 2 h with SCF, dasatinib or midostaurin were analysed by immunoblotting with the indicated antibodies. The illustration indicates blockage of KIT receptor activity by TKIs. (b) sEV release from HMC‐1.2 cells treated or not with dasatinib or midostaurin. Data are represented as the mean ± SD from three independent experiments. (c) Average size (nm) of sEVs secreted by untreated or inhibitor‐treated HMC‐1.2 cells. Plotted data are shown from three independent experiments and lines are connecting the datapoints for each experiment. (d) Size distribution in percentage of HMC‐1.2‐derived sEVs from untreated, or KIT inhibitor‐treated cells. Shown data represent the mean from three independent experiments. (e) Immunoblot analysis of HMC‐1.2 sEV lysates. Representative blots are shown. Equal EV protein (20 μg) was loaded. The indicated antibodies were used for signal detection and grouped in typical exosome or ectosome representing markers as highlighted. Dashed lines indicate blots were from the same membrane but discontinuously loaded. ***, *p* < 0.001; ****, *p* < 0.0001 (unpaired *t* test). sEV, small extracellular vesicle; TKI, tyrosine kinase inhibitor.

We then treated HMC‐1.2 cells with the tyrosine kinase inhibitors dasatinib and midostaurin. These inhibitors effectively blocked the constitutive activity of KIT as evident by the absence of KIT phosphorylation (Figure 3a). Of note, the secretion of HMC‐1.2 sEVs was significantly reduced by 50% when KIT activity was inhibited (Figure 3b). The average sEV size did not change as we saw previously after SCF stimulation (Figure 3c), but the proportion of the smallest EVs (0–100 nm) declined (Figure 3d). Concomitant with the decrease in quantity, exosome protein markers such as syntenin‐1 and CD63 were reduced in sEVs produced from KIT inhibitor‐treated cells, while other markers (ARF6, basigin) did not markedly change (Figure 3e). These findings demonstrate that activated KIT promotes the release of sEVs in a regulated fashion, as the high secretion of exosome‐like sEVs by neoplastic huMCs is partially reversed when KIT activity is blocked.

### 3.3 KIT signalling cascades contribute to the release of sEVs

A comparative analysis of transcriptomic data obtained from HMC‐1.1 and HMC‐1.2 cells (Bandara et al., 2023) revealed that endocytic processes were significantly upregulated in HMC‐1.2 cells (Figure S4). Since KIT is highly phosphorylated in HMC‐1.2 cells (Figure 1a) and RTKs are endocytosed after activation, we sought to examine whether internalisation of KIT was required for the stimulated exosome‐like sEV release. Inhibition of clathrin‐mediated endocytosis by Pitstop2 in HMC‐1.2 cells had a small impact on sEV numbers (Figure S5a,b). Dynasore, a dynamin inhibitor that blocks clathrin‐independent endocytosis, significantly reduced the number of secreted sEVs. However, it also affected the phosphorylation status of KIT (Figure S5a,b). Additionally, sEVs secreted from dynasore‐treated cells showed reduced expression of exosome protein markers such as syntenin‐1, CD63 or ALIX (Figure S5c). Thus, although receptor internalisation of KIT may be involved in the stimulated generation or release of sEVs, the result cannot be dissociated from the effect on KIT phosphorylation.

**FIGURE 4:**
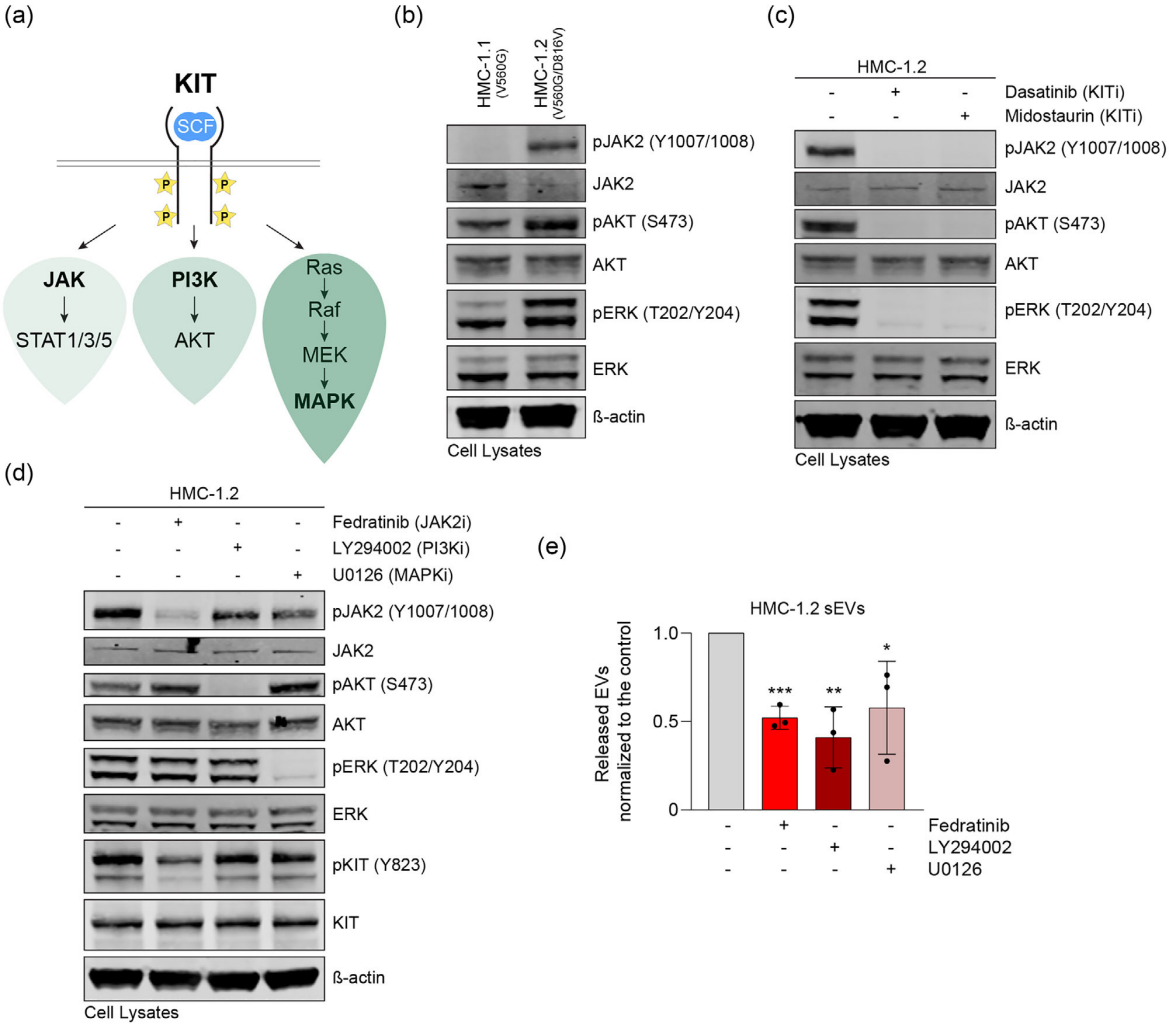
Oncogenic KIT signalling cascades contribute to the secretion of sEVs. (a) Diagram of the receptor KIT and simplified signalling pathways stimulated in response to the activation of KIT. (b) Comparison of expression levels of signalling proteins in HMC‐1.1 and HMC‐1.2 cells. Equal protein (30 μg) was loaded. (c) Lysates (30 μg of protein) of HMC‐1.2 cells treated or not with the KIT inhibitors dasatinib or midostaurin were analysed for the activation status of signalling kinases with the indicated phospho‐antibodies. Blots were also probed with the indicated antibodies recognizing the corresponding total protein. (d) HMC‐1.2 cells were tested for the specificity of JAK2 (Fedratinib), PI3K (LY294002) and MAPK (U0126) inhibitors by Western blotting with the indicated antibodies. Equal protein (30 μg) was loaded. (e) Secretion of sEVs from HMC‐1.2 cells after treatment with indicated signalling pathway inhibitors. Data are represented as the mean ± SD from three independent experiments. *, *p* < 0.05; **, *p* < 0.01; ***, *p* < 0.001 (unpaired *t* test). sEV, small extracellular vesicle.

We also considered that early signalling triggered by KIT may play a role in the regulation of sEV release. The activation of KIT stimulates several downstream pathways including the JAK, PI3K and MAPK signalling cascades (Figure 4a). While HMC‐1.1 cells contain one mutation in KIT (V560G), HMC‐1.2 cells express two KIT mutations (V560G and D816V). As a result, constitutive, oncogenic KIT signalling (pJAK2, pAKT, pERK) is enhanced in HMC‐1.2 cells compared to HMC‐1.1 cells (Figure 4b) (Bandara et al., 2023). In the presence of KIT inhibitors, the activation of these downstream signalling proteins was equally blocked (Figure 4c). To decipher which pathways may contribute to the secretion of sEVs, we used specific inhibitors targeting JAK2 (fedratinib), PI3K (LY294002) or MAPK (U0126). While the phosphorylation of their respective targets was drastically reduced, immunoblots did not reveal either any cross‐inhibitory effects on other pathways or inhibited KIT receptor phosphorylation (Figure 4d). HMC‐1.2 cells that were incubated with JAK2, PI3K or MAPK inhibitors showed a significantly reduced secretion of sEVs (Figure 4e). Together, these data are consistent with the conclusion that several early signalling pathways induced by KIT activation contribute to the regulated release of sEVs by neoplastic huMCs.

### 3.4 SCF‐induced activation of normal KIT in huMCs stimulates exosome‐like sEV secretion without inducing degranulation

**FIGURE 5:**
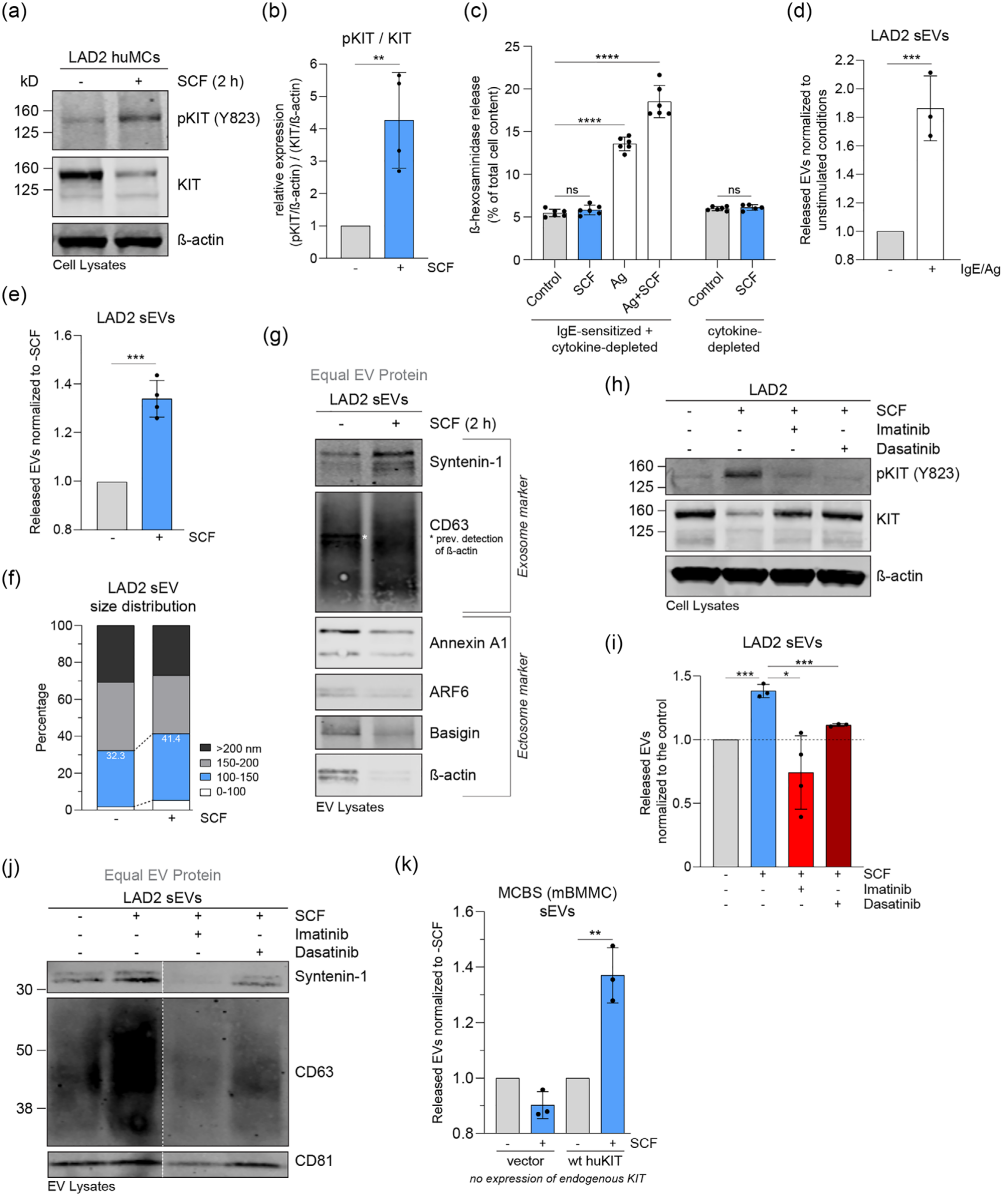
SCF stimulation of normal KIT expressed by LAD2 huMCs triggers enhanced sEV secretion. (a) LAD2 huMCs expressing normal KIT were stimulated or not with the receptor ligand SCF. Cell lysates (30 μg of protein) were analysed by immunoblotting with the indicated antibodies. (b) Quantification of KIT phosphorylation (pKIT) in LAD2 cells treated or not with SCF (A). The relative expression of pKIT/KIT was normalised to non‐stimulated cells. Data are represented as the mean ± SD from four independent experiments. (c) Analysis of degranulation activity of LAD2 huMCs by measurement of released β‐hexosaminidase. Cells cultured overnight in cytokine‐depleted media with or without antigen‐specific IgE were stimulated by SCF or antigen (Ag) the next day. Data are represented as the mean ± SD from three independent experiments; each experiment was run in duplicates. (d) Secreted sEVs from LAD2 huMCs activated or not by IgE/antigen. IgE priming was performed overnight in regular SCF‐containing culture media. Data are shown as the mean ± SD from three independent experiments. (e) sEV release over 2 h by LAD2 huMCs stimulated or not with SCF. Plotted data are shown as the mean ± SD from four independent experiments. (f) Size distribution in percentage of sEVs released from LAD2 cells treated or not with SCF. The percentage of EVs smaller than 150 nm is highlighted. Shown data represent the mean from four independent experiments. (g) Immunoblot analysis of LAD2‐derived sEV lysates. Representative blots of three independent experiments are shown. Equal EV protein (20 μg) was loaded. The indicated antibodies were used for signal detection and grouped into classical exosome or ectosome markers as highlighted. (h) Cytokine‐depleted LAD2 cells were incubated with SCF, or SCF plus the KIT inhibitors imatinib or dasatinib. Cell lysates (30 μg of protein) were analysed by Western blotting with the indicated antibodies. (i) sEV release from LAD2 huMCs treated or not with SCF and the KIT inhibitors imatinib or dasatinib. Data are represented as the mean ± SD from three to four independent experiments. (j) Immunoblot analysis of LAD2 sEV lysates. Representative blots of three experiments are shown. Equal EV protein (20 μg) was loaded and analysed by the indicated antibodies. Dashed lines indicate blots were from the same membrane but discontinuously loaded. (k) sEV secretion by murine bone marrow mast cells (mBMMCs) that do not express endogenous KIT and were stably transfected with empty vector or normal human KIT (huKIT) (MCBS cell lines). sEVs were isolated from control or SCF‐treated cells. Data are represented as the mean ± SD from three independent experiments. *, *p* < 0.05; **, *p* < 0.01; ***, *p* < 0.001; ****, *p* < 0.0001 (unpaired *t* test). Ns, not significant; sEV, small extracellular vesicle.

We further sought to examine stimulated sEV secretion by activated, non‐mutated KIT. To this end, we employed normal KIT expressing LAD2 huMCs in which the phosphorylation of KIT is strictly controlled by SCF (Figure 5a,b). Since it has been reported that murine MCs co‐secrete EVs during degranulation (Groot Kormelink et al., 2016; Liang et al., 2020; Molfetta et al., 2020), we first tested for potential MC degranulation induced by SCF under our experimental conditions (Figure 5c). While MCs degranulated in the presence of antigen, we did not detect additional activation above the baseline level when LAD2 cells were stimulated with SCF to activate KIT (Figure 5c). Notably, LAD2 huMCs secreted twice as many sEVs after activation by IgE/antigen (Figure 5d), which confirms previous studies conducted in murine and rat MCs (Groot Kormelink et al., 2016; Liang et al., 2020; Molfetta et al., 2020; Raposo et al., 1997). SCF induced the secretion of significantly more sEVs compared to non‐stimulated LAD2 cells (Figure 5e), emphasizing a role for KIT activation in the release of sEVs. Compared to unstimulated cells, the proportion of sEVs smaller than 150 nm increased after SCF‐mediated KIT activation (Figure 5f). Similar to HMC‐1 cells, stimulation with SCF increased exosome protein markers (syntenin‐1, CD63) in sEVs and reduced ectosome markers (Figure 5g). These results indicate that activated KIT stimulates the secretion of exosome‐like sEVs in huMCs expressing both oncogenic and normal KIT. Moreover, the SCF‐induced release of sEVs occurs independently of MC degranulation.

To probe into the specificity of the SCF‐triggered secretion of sEVs, we tested the effect of KIT inhibitors on the sEV release from LAD2 huMCs. Imatinib and dasatinib, which efficiently inhibited SCF‐induced activation of KIT in LAD2 huMCs (Figure 5h), dampened the increase in the sEV release (Figure 5i) and prevented the increase of exosome protein markers (syntenin‐1, CD63) in SCF‐activated sEVs (Figure 5j). Additionally, we used a murine MC model (MCBS‐1) that lacks endogenous KIT expression but stably expresses human KIT. MCBS‐1 cells expressing human KIT, but not the vector control cell line, responded to SCF by increasing the secretion of sEVs (Figure 5k). In summary, these findings demonstrate that KIT activation promotes the release of sEVs in a specific manner, as the stimulated sEV secretion is reversed when KIT activity is blocked.

### 3.5 Stimulation of EGFR in HeLa cells does not trigger enhanced sEV secretion

**FIGURE 6:**
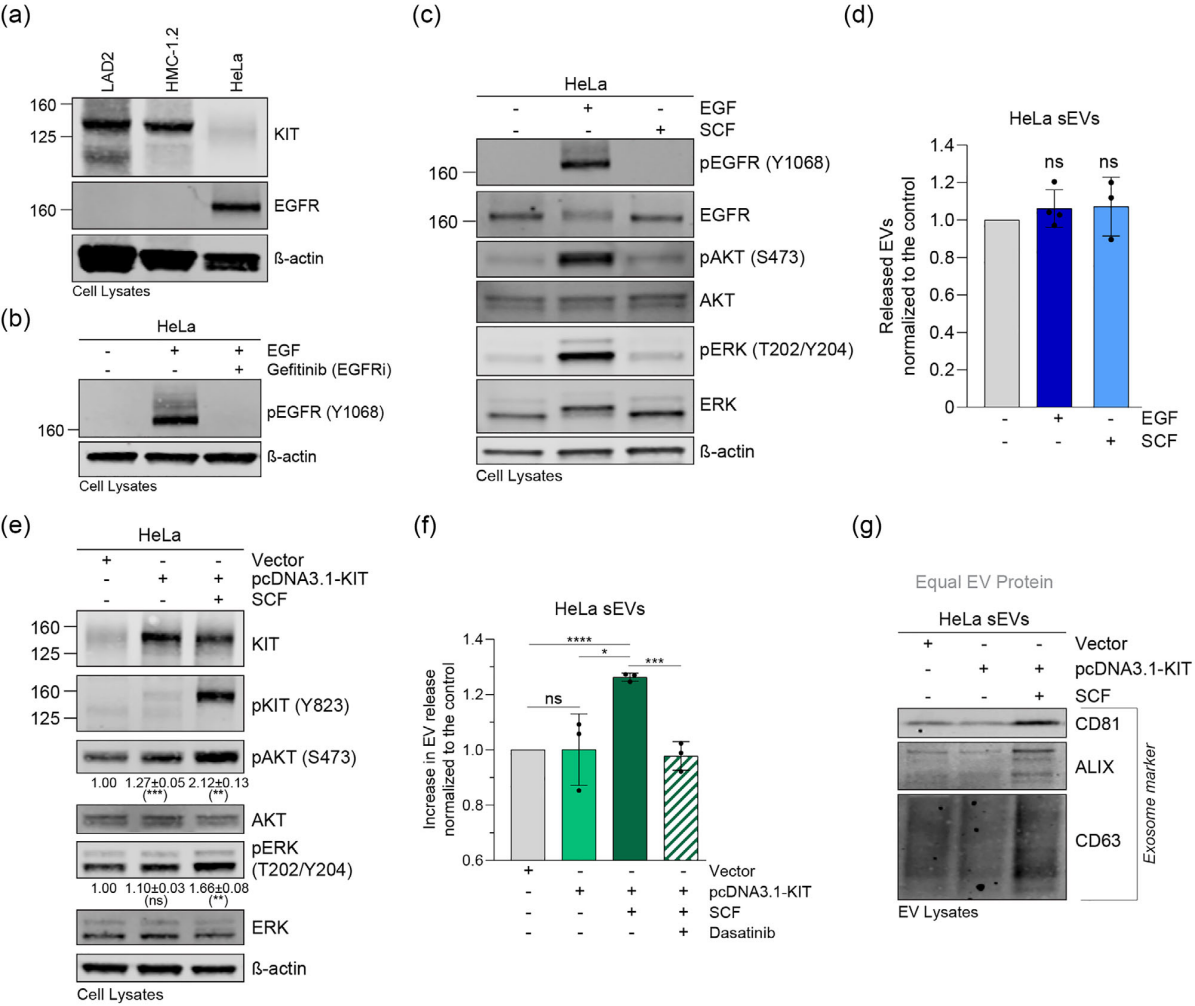
Enhanced sEV secretion is a general consequence of KIT activation but not of EGFR stimulation. (a) LAD2, HMC‐1.2 and HeLa cell lysates were compared for expression levels of KIT and EGFR by immunoblotting. Equal protein (30 μg) was loaded. (b) HeLa cells were treated or not with EGF to activate EGFR in the presence or absence of the EGFR inhibitor gefitinib. Activation of EGFR was confirmed by Western blotting. Equal protein (30 μg) was loaded. (c) Lysates (30 μg of protein) of HeLa cells incubated for 10 min with EGF or SCF were analysed for activation of EGFR and signalling proteins by immunoblotting with the indicated antibodies. (d) sEV secretion by HeLa cells stimulated with EGF or SCF for 2 h. Data are represented as the mean ± SD from three to four independent experiments. (e) HeLa cells were transiently transfected with empty pcDNA3.1 vector or KIT‐encoding pcDNA3.1 vector. At 24 h post‐transfection, KIT was activated or not by SCF. Cell lysates (30 μg of protein) were analysed by Western blotting with the indicated antibodies. The relative increase of pAKT and pERK was normalised to empty vector‐transfected cells and indicated below the immunoblots. Expression data are represented as the mean ± SD from three independent experiments. (f) sEV secretion by HeLa cells in (e). sEVs were also quantified after KIT activation in KIT inhibitor (dasatinib) treated cells. Data are represented as the mean ± SD from three independent experiments. (g) Immunoblot analysis of HeLa sEVs in (f) with the indicated antibodies. Equal EV protein (20 μg) was loaded. *, *p* < 0.05; **, *p* < 0.01; ***, *p* < 0.001; ****, *p* < 0.0001 (unpaired *t* test). EGFR, epidermal growth factor receptor; EV, extracellular vesicles; Ns, not significant; sEV, small extracellular vesicle; SCF, stem cell factor.

Since the activity status of KIT in MCs influences the quantity of secreted sEVs, we reasoned that the stimulation of other RTKs with shared signalling pathways to KIT may have similar effects. While the widely studied RTK EGFR is not expressed in MCs, HeLa epithelial cells express endogenous EGFR but not KIT (Figure 6a). As expected, EGF induced the phosphorylation of its receptor EGFR, which was prevented in the presence of the EGFR inhibitor gefitinib, demonstrating functionality of EGFR and specificity of the EGF stimulation in HeLa cells (Figure 6b). Furthermore, EGF, but not SCF, activated EGFR downstream signalling such as the MAPK and PI3K pathways (Figure 6c). However, activation of EGFR in HeLa cells did not enhance the secretion of sEVs as seen for activated KIT in MCs (Figure 6d). While we only analysed EGFR, these results suggest that enhanced cell secretion of sEVs is not a general feature of activated RTKs and could be cell type dependent.

### 3.6 Activation of transiently expressed KIT stimulates enhanced sEV secretion in HeLa cells

To address whether activation of KIT could enhance sEV secretion in cells other than MCs, we transfected HeLa cells with wild‐type KIT and tested for potential SCF‐triggered release of sEVs. SCF‐induced phosphorylation of transiently expressed KIT in HeLa cells activated downstream protein kinases (Figure 6e), suggesting proper coupling to KIT signalling cascades. Concomitantly, SCF induced a significant increase in the secretion of sEVs in KIT‐transfected HeLa cells, but not in cells transfected with the empty vector, and, additionally, the stimulated sEV release was prevented in the presence of the KIT inhibitor dasatinib (Figure 6f). HeLa sEVs induced by SCF also expressed increased levels of typical exosome protein markers (CD81, ALIX, CD63), as shown in huMCs, pointing towards a potential exosomal origin (Figure 6g). In conclusion, our data demonstrate that KIT signalling induces sEV release in huMCs and HeLa cells and that early signalling cascades partially orchestrate the sEV release.

## 4 DISCUSSION

A collection of studies has established that cells dynamically modify the quantity and molecular composition of secreted EVs in response to activating stimuli (Groot Kormelink et al., 2016; Nolte‐’t Hoen et al., 2013; Segura et al., 2005; van der Vlist et al., 2012; Verweij et al., 2018). Our work here expands the existing literature by providing an example of how the inducible secretion of sEVs can be regulated by receptor signalling as opposed to the constitutive release of vesicles. Specifically, we unveiled a functional correlation between the quantity and quality of secreted sEVs and the activity status of the receptor KIT (Figure S6). The stimulated sEV release was reversed by KIT inhibitors or inhibition of signalling molecules downstream of KIT activation and, thus, this study highlights that the secretion of sEVs can be controlled by cellular pathways that may stimulate or reduce the release. Furthermore, in MCs, induced EV secretion has been described to co‐occur with degranulation, and we elucidated here that SCF‐induced signalling leading to the secretion of exosome‐like sEVs is uncoupled from granular extrusion, suggesting there are MC activating stimuli that distinctively trigger the release of sEVs without targeting the exocytosis of granules. The finding that sEV secretion is stimulated by signalling cascades that also transduce functional responses by activated KIT provides impetus to the concept of EVs being part of KIT‐associated biological functions in the microenvironment and/or regulation of KIT availability and signalling.

Neoplastic MCs expressing constitutively active KIT have been recognised to secrete substantial quantities of EVs. In addition, patients with oncogenic KIT‐driven diseases have higher concentrations of circulating EVs than healthy controls (Atay et al., 2018; Kim et al., 2021, 2018). Here, seeking an explanation for these observations, we investigated a potential role for KIT receptor activation in regulating EV secretion. Our study demonstrates that constitutive KIT activation caused by gain‐of‐function mutations, and ligand‐mediated KIT activation, induce the release of sEVs in a KIT activity‐ and signalling‐dependent manner. The data reveal that typical exosome markers such as syntenin‐1 and CD63 were upregulated in sEVs in correlation with increased KIT activity, while ectosome markers were downregulated. Therefore, we suspect that a majority of the sEVs released during KIT activation are exosome‐like. The differences in EV markers in the context of KIT activation raise the possibility of other accompanying changes in the molecular composition of sEVs, potentially with functional consequences in the in vivo environment in KIT‐related diseases. This is supported by the proposed role of circulating EVs in the pathogenesis of oncogenic KIT‐driven diseases where EVs may alter the phenotypes of distal bone (Kim et al., 2021), and liver cells (Kim et al., 2018); and by the effects EVs released from oncogenic KIT‐expressing cells have on numerous cell types (Al‐Nedawi et al., 2005; Atay et al., 2014; Elsemüller et al., 2019; Shelke et al., 2019; Xiao et al., 2014; Yin et al., 2020). EV‐mediated transfer of KIT protein and subsequent activation of signalling pathways in recipient cells have been reported to contribute to these effects (Atay et al., 2014; Kim et al., 2018; Xiao et al., 2014). sEVs secreted from SCF‐stimulated MCs contained significantly increased ratios of phosphorylated to total KIT (Figure S7a,b). It remains to be determined whether the transfer of more phosphorylated KIT by sEVs will trigger more effectively the signalling cascades and functional/phenotypic changes in recipient cells than the EV‐transfer of non‐phosphorylated KIT. Finally, our demonstration that the KIT‐induced sEV release is blocked by KIT inhibitors is consistent with the reported reduced numbers of EVs and disease markers in responsive GIST patients after treatment with the KIT inhibitor imatinib (Atay et al., 2018) and poses the question of whether blockage of EV release could partly contribute to the benefits of treatment.

The enhanced secretion of exosome‐like sEVs induced by KIT activation may have several potential reasons, including (i) the generation of more endosomes due to stimulated receptor internalisation by endocytosis, (ii) enhanced ILV production leading to more vesicles per MVB, and/or (iii) enhanced fusion rate of MVBs with the plasma membrane. We considered that receptor internalisation may be a contributing factor as this process is driven by endocytosis and causes a potential quantitative increase of endosomes, which are the ultimate source of exosomes. We addressed this by using endocytosis inhibitors and observed decreased sEV secretion. However, the phosphorylation of KIT was simultaneously reduced so that we cannot conclude whether the reduction in sEV secretion was due to perturbed KIT internalisation or decreased receptor activity. EM imaging of thin sections revealed that HMC‐1.2 cells contain fewer MVBs per cytoplasmic area than HMC‐1.1 cells. This observation is consistent with the possibility that increased KIT activity and signalling lead to an increased fusion rate of MVBs with the plasma membrane resulting in the enhanced secretion of exosome‐like sEVs, similar to what has been reported for the stimulation of the histamine H1 receptor (Verweij et al., 2018), and which will need additional investigation going forward.

The molecular mechanisms regulating enhanced EV secretion from activated cells are not fully understood and remain largely vague. Cell activation can evoke an increase of intracellular calcium concentrations leading to the disintegration of the plasma membrane asymmetry, which supports membrane blebbing and the formation of ectosomes (Hugel et al., 2005). Exosome release requires the transport, docking, and fusion of MVBs with the plasma membrane orchestrated by RAB, SNARE and cytoskeletal proteins and this process may be calcium‐dependent (Mathieu et al., 2019; Messenger et al., 2018; Savina et al., 2003; Xu et al., 2022). However, there are also examples that calcium signalling may be dispensable for the release of exosomes. For instance, activation of the G protein‐coupled receptor histamine H1 in HeLa cells resulted in increased phosphorylation of SNAP23, which promoted the fusion of MVBs with the plasma membrane and the secretion of CD63‐containing exosomes in a calcium‐independent fashion (Verweij et al., 2018). Several extracellular stimuli have been identified that may contribute to triggering and regulating EV biogenesis (Jin et al., 2022). However, the exact regulatory mechanisms and signalling pathways that define the controlled secretion of EVs, in terms of triggering and ending a stimulated release, remain largely undefined and are just beginning to be elucidated (Jin et al., 2023; Lee et al., 2023; Wang et al., 2023; Wu et al., 2023).

Adding to these studies, we show that in HMC‐1.2 cells the activation of pathways downstream of oncogenic KIT, including PI3K, JAK and MAPK signalling, contribute to the regulated release of sEVs. Mutant KIT accumulates in endo‐lysosomal compartments and as seen for other activated RTKs, can remain associated to signalling molecules, which continue to signal in these compartments (Critchley et al., 2018; Cruse et al., 2014; Obata et al., 2014). However, how these early signals intersect with cytoskeletal rearrangements and/or assembly of complex apparatuses for MVB formation, trafficking, and fusion needs further investigation. A potential link between PI3K and EV release may relate to its role in the activation of mTORC, which is upregulated in HMC‐1.2 cells (Smrz et al., 2011), and has been implicated in EV release via autophagy depression or inhibition of the lysosomal route, thus promoting MVB formation and exosome release (Mitani et al., 2022; Ryskalin et al., 2020). Similarly, other models for the crosstalk between endocytosis and cell signalling have been proposed where impairment of MVB fusion with the lysosome can upregulate the fusion rate of MVBs with the plasma membrane resulting in enhanced exosome secretion (Dobrowolski & De Robertis, 2011; Shelke et al., 2023). Therefore, future studies will be needed to dissect whether KIT‐specific signalling pathways regulate endo‐lysosomal fusion events to stimulate the secretion of exosome‐like sEVs. Although signalling pathways are generally shared by different receptor types and specifically between RTKs, activation of EGFR in HeLa cells surprisingly did not result in enhanced sEV release, indicating that triggering the secretion of sEVs is not a general hallmark of activated RTKs. However, the transient expression of KIT in HeLa cells drove an enhanced release of sEVs after SCF‐mediated activation. While many signalling pathways are shared by various receptors, activation of prominent signalling proteins alone is not the mere key to triggering sEV secretion. We speculate there are more KIT‐specific signalosome complexes driving sEV secretion that we do not understand yet, warranting further investigation. Beyond the cellular release of exosome‐like sEVs, it is also tempting to speculate that MVBs contained within large multi‐compartmented microvesicles (MCMVs) may be triggered to fuse with the MCMV membrane to release exosomes, when receptors on MCMVs encounter soluble or cell‐bound ligands (Petersen et al., 2023).

In conclusion, we have identified that activation of the receptor KIT triggers an enhanced and regulated secretion of exosome‐like sEVs. These findings complement previous reports showing that activated cells dynamically modify the quantity and quality of secreted sEVs and extend our understanding of potential regulatory mechanisms that define the controlled sEV release in response to activating stimuli. The results from this study will encourage further investigation into the composition and biological functions of MC‐derived EVs released in response to KIT activation or other stimuli.

## AUTHOR CONTRIBUTIONS

**Annika Pfeiffer**: Conceptualisation; Methodology; Investigation; Writing—original draft; Writing—review and editing; Visualisation; Project administration. **Ana Olivera**: Conceptualisation; Methodology; Writing—original draft; Writing—review and editing; Supervision; Project administration. **Jennifer D. Petersen**: Methodology; Investigation; Writing—review and editing. **Geethani Bandara**: Investigation; Writing—review and editing. **Guido H. Falduto**: Investigation; Writing—review and editing. **Dean D. Metcalfe**: Supervision; Writing—review and editing **Joshua Zimmerberg**: Supervision; Writing—review and editing.

## CONFLICT OF INTEREST STATEMENT

The authors report no conflict of interest.

## Supporting information

Supporting Information

## Data Availability

Data are provided in this article and are available from the corresponding author upon reasonable request.

## References

[jex2139-bib-0001] Al‐Nedawi, K. , Szemraj, J. , & Cierniewski, C. S. (2005). Mast cell‐derived exosomes activate endothelial cells to secrete plasminogen activator inhibitor type 1. Arteriosclerosis, Thrombosis, and Vascular Biology, 25, 1744–1749. 15920032 10.1161/01.ATV.0000172007.86541.76

[jex2139-bib-0002] Atay, S. , Banskota, S. , Crow, J. , Sethi, G. , Rink, L. , & Godwin, A. K. (2014). Oncogenic KIT‐containing exosomes increase gastrointestinal stromal tumor cell invasion. PNAS, 111, 711–716. 24379393 10.1073/pnas.1310501111 PMC3896203

[jex2139-bib-0003] Atay, S. , Wilkey, D. W. , Milhem, M. , Merchant, M. , & Godwin, A. K. (2018). Insights into the proteome of gastrointestinal stromal tumors‐derived exosomes reveals new potential diagnostic biomarkers. Molecular & Cellular Proteomics, 17, 495–515. 29242380 10.1074/mcp.RA117.000267 PMC5836374

[jex2139-bib-0004] Bandara, G. , Falduto, G. H. , Luker, A. , Bai, Y. , Pfeiffer, A. , Lack, J. , Metcalfe, D. D. , & Olivera, A. (2023). CRISPR/Cas9‐engineering of HMC‐1.2 cells renders a human mast cell line with a single D816V‐KIT mutation: An improved preclinical model for research on mastocytosis. Frontiers in Immunology, 14, 1078958. 37025992 10.3389/fimmu.2023.1078958 PMC10071028

[jex2139-bib-0005] Blanchard, N. , Lankar, D. , Faure, F. , Regnault, A. , Cl, D. , Ga, R. , & Hivroz, C. (2002). TCR activation of human T cells induces the production of exosomes bearing the TCR/CD3/ζ complex^1^ . The Journal of Immunology, 168, 3235–3241. 11907077 10.4049/jimmunol.168.7.3235

[jex2139-bib-0006] Butterfield, J. H. , Weiler, D. , Dewald, G. , & Gleich, G. J. (1988). Establishment of an immature mast cell line from a patient with mast cell leukemia. Leukemia Research, 12, 345–355. 3131594 10.1016/0145-2126(88)90050-1

[jex2139-bib-0007] Chan, E. C. , Bai, Y. , Bandara, G. , Simakova, O. , Brittain, E. , Scott, L. , Dyer, K. D. , Klion, A. D. , Maric, I. , Gilfillan, A. M. , Metcalfe, D. D. , & Wilson, T. M. (2013). KIT GNNK splice variants: Expression in systemic mastocytosis and influence on the activating potential of the D816V mutation in mast cells. Experimental Hematology, 41, 870–881. 23743299 10.1016/j.exphem.2013.05.005 PMC3816383

[jex2139-bib-0008] Clancy, J. W. , Zhang, Y. , Sheehan, C. , & D'Souza‐Schorey, C. (2019). An ARF6‐Exportin‐5 axis delivers pre‐miRNA cargo to tumour microvesicles. Nature Cell Biology, 21, 856–866. 31235936 10.1038/s41556-019-0345-y PMC6697424

[jex2139-bib-0009] Critchley, W. R. , Pellet‐Many, C. , Ringham‐Terry, B. , Harrison, M. A. , Zachary, I. C. , & Ponnambalam, S. (2018). Receptor tyrosine kinase ubiquitination and de‐ubiquitination in signal transduction and receptor trafficking. Cells, 7(3), 22. 29543760 10.3390/cells7030022 PMC5870354

[jex2139-bib-0010] Cruse, G. , Metcalfe, D. D. , & Olivera, A. (2014). Functional deregulation of KIT: Link to mast cell proliferative diseases and other neoplasms. Immunology and Allergy Clinics of North America, 34, 219–237. 24745671 10.1016/j.iac.2014.01.002 PMC3994404

[jex2139-bib-0011] Dobrowolski, R. , & De Robertis, E. M. (2011). Endocytic control of growth factor signalling: Multivesicular bodies as signalling organelles. Nature Reviews Molecular Cell Biology, 13, 53–60. 22108513 10.1038/nrm3244 PMC3374592

[jex2139-bib-0012] Elsemüller, A. K. , Tomalla, V. , Gärtner, U. , Troidl, K. , Jeratsch, S. , Graumann, J. , Baal, N. , Hackstein, H. , Lasch, M. , Deindl, E. , Preissner, K. T. , & Fischer, S. (2019). Characterization of mast cell‐derived rRNA‐containing microvesicles and their inflammatory impact on endothelial cells. FASEB Journal, 33, 5457–5467. 30702929 10.1096/fj.201801853RR

[jex2139-bib-0013] Falduto, G. H. , Pfeiffer, A. , Luker, A. , Metcalfe, D. D. , & Olivera, A. (2021). Emerging mechanisms contributing to mast cell‐mediated pathophysiology with therapeutic implications. Pharmacology & Therapeutics, 220, 107718. 33130192 10.1016/j.pharmthera.2020.107718

[jex2139-bib-0014] Groot Kormelink, T. , Arkesteijn, G. J. , van de Lest, C. H. , Geerts, W. J. , Goerdayal, S. S. , Altelaar, M. A. , Redegeld, F. A. , Nolte‐’t Hoen, E. N. , & Wauben, M. H. (2016). Mast cell degranulation is accompanied by the release of a selective subset of extracellular vesicles that contain mast cell‐specific proteases. Journal of Immunology, 197, 3382–3392. 10.4049/jimmunol.1600614 27619994

[jex2139-bib-0015] Hugel, B. , Martínez, M. C. , Kunzelmann, C. , & Freyssinet, J. M. (2005). Membrane microparticles: Two sides of the coin. Physiology, 20, 22–27. 15653836 10.1152/physiol.00029.2004

[jex2139-bib-0016] Hundley, T. R. , Gilfillan, A. M. , Tkaczyk, C. , Andrade, M. V. , Metcalfe, D. D. , & Beaven, M. A. (2004). Kit and FcepsilonRI mediate unique and convergent signals for release of inflammatory mediators from human mast cells. Blood, 104, 2410–2417. 15217825 10.1182/blood-2004-02-0631

[jex2139-bib-0017] Jeppesen, D. K. , Fenix, A. M. , Franklin, J. L. , Higginbotham, J. N. , Zhang, Q. , Zimmerman, L. J. , Liebler, D. C. , Ping, J. , Liu, Q. , & Evans, R. (2019). Reassessment of exosome composition. Cell, 177, 428–445. 30951670 10.1016/j.cell.2019.02.029 PMC6664447

[jex2139-bib-0018] Jin, X. , Xia, T. , Luo, S. , Zhang, Y. , Xia, Y. , & Yin, H. (2023). Exosomal lipid PI4P regulates small extracellular vesicle secretion by modulating intraluminal vesicle formation. Journal of Extracellular Vesicles, 12, e12319. 37021404 10.1002/jev2.12319 PMC10076970

[jex2139-bib-0019] Jin, Y. , Ma, L. , Zhang, W. , Yang, W. , Feng, Q. , & Wang, H. (2022). Extracellular signals regulate the biogenesis of extracellular vesicles. Biological Research, 55, 35. 36435789 10.1186/s40659-022-00405-2 PMC9701380

[jex2139-bib-0020] Kim, D. K. , Bandara, G. , Cho, Y. E. , Komarow, H. D. , Donahue, D. R. , Karim, B. , Baek, M. C. , Kim, H. M. , Metcalfe, D. D. , & Olivera, A. (2021). Mastocytosis‐derived extracellular vesicles deliver miR‐23a and miR‐30a into pre‐osteoblasts and prevent osteoblastogenesis and bone formation. Nature Communications, 12, 2527. 10.1038/s41467-021-22754-4 PMC8100305 33953168

[jex2139-bib-0021] Kim, D. K. , Cho, Y. E. , Komarow, H. D. , Bandara, G. , Song, B. J. , Olivera, A. , & Metcalfe, D. D. (2018). Mastocytosis‐derived extracellular vesicles exhibit a mast cell signature, transfer KIT to stellate cells, and promote their activation. PNAS, 115, E10692–E10701. 30352845 10.1073/pnas.1809938115 PMC6233074

[jex2139-bib-0022] Kirshenbaum, A. S. , Akin, C. , Wu, Y. , Rottem, M. , Goff, J. P. , Beaven, M. A. , Rao, V. K. , & Metcalfe, D. D. (2003). Characterization of novel stem cell factor responsive human mast cell lines LAD 1 and 2 established from a patient with mast cell sarcoma/leukemia; activation following aggregation of FcepsilonRI or FcgammaRI. Leukemia Research, 27, 677–682. 12801524 10.1016/s0145-2126(02)00343-0

[jex2139-bib-0023] Kowal, J. , Arras, G. , Colombo, M. , Jouve, M. , Morath, J. P. , Primdal‐Bengtson, B. , Dingli, F. , Loew, D. , Tkach, M. , & Théry, C. (2016). Proteomic comparison defines novel markers to characterize heterogeneous populations of extracellular vesicle subtypes. PNAS, 113, E968–E977. 26858453 10.1073/pnas.1521230113 PMC4776515

[jex2139-bib-0024] Kuehn, H. S. , Radinger, M. , & Gilfillan, A. M. (2010). Measuring mast cell mediator release. Current protocols in immunology, 91, 7.38.1–7.38.9. 10.1002/0471142735.im0738s91 PMC2982193 21053305

[jex2139-bib-0025] Kugeratski, F. G. , Hodge, K. , Lilla, S. , McAndrews, K. M. , Zhou, X. , Hwang, R. F. , Zanivan, S. , & Kalluri, R. (2021). Quantitative proteomics identifies the core proteome of exosomes with syntenin‐1 as the highest abundant protein and a putative universal biomarker. Nature Cell Biology, 23, 631–641. 34108659 10.1038/s41556-021-00693-y PMC9290189

[jex2139-bib-0026] Lee, Y. J. , Shin, K. J. , Jang, H. J. , Ryu, J. S. , Lee, C. Y. , Yoon, J. H. , Seo, J. K. , Park, S. , Lee, S. , Je, A. R. , Huh, Y. H. , Kong, S. Y. , Kwon, T. , Suh, P. G. , & Chae, Y. C (2023). GPR143 controls ESCRT‐dependent exosome biogenesis and promotes cancer metastasis. Developmental Cell, 58, 320–334. 36800996 10.1016/j.devcel.2023.01.006

[jex2139-bib-0027] Liang, Y. , Huang, S. , Qiao, L. , Peng, X. , Li, C. , Lin, K. , Xie, G. , Li, J. , Lin, L. , Yin, Y. , Liao, H. , Li, Q. , & Li, L. (2020). Characterization of protein, long noncoding RNA and microRNA signatures in extracellular vesicles derived from resting and degranulated mast cells. Journal of Extracellular Vesicles, 9, 1697583. 31853339 10.1080/20013078.2019.1697583 PMC6913652

[jex2139-bib-0028] Mathieu, M. , Martin‐Jaular, L. , Lavieu, G. , & Théry, C. (2019). Specificities of secretion and uptake of exosomes and other extracellular vesicles for cell‐to‐cell communication. Nature Cell Biology, 21, 9–17. 30602770 10.1038/s41556-018-0250-9

[jex2139-bib-0029] Mathieu, M. , Névo, N. , Jouve, M. , Valenzuela, J. I. , Maurin, M. , Verweij, F. J. , Palmulli, R. , Lankar, D. , Dingli, F. , Loew, D. , Rubinstein, E. , Boncompain, G. , Perez, F. , & Théry, C. (2021). Specificities of exosome versus small ectosome secretion revealed by live intracellular tracking of CD63 and CD9. Nature Communications, 12, 4389. 10.1038/s41467-021-24384-2 PMC8289845 34282141

[jex2139-bib-0030] Messenger, S. W. , Woo, S. S. , Sun, Z. , & Martin, T. F. J. (2018). A Ca^2+^‐stimulated exosome release pathway in cancer cells is regulated by Munc13‐4. Journal of Cell Biology, 217, 2877–2890. 29930202 10.1083/jcb.201710132 PMC6080937

[jex2139-bib-0031] Mitani, F. , Lin, J. , Sakamoto, T. , Uehara, R. , Hikita, T. , Yoshida, T. , Setiawan, A. , Arai, M. , & Oneyama, C. (2022). Asteltoxin inhibits extracellular vesicle production through AMPK/mTOR‐mediated activation of lysosome function. Scientific Reports, 12, 6674. 35461323 10.1038/s41598-022-10692-0 PMC9035176

[jex2139-bib-0032] Molfetta, R. , Lecce, M. , Quatrini, L. , Caracciolo, G. , Digiacomo, L. , Masuelli, L. , Milito, N. D. , Vulpis, E. , Zingoni, A. , Galandrini, R. , Santoni, A. , & Paolini, R. (2020). Immune complexes exposed on mast cell‐derived nanovesicles amplify allergic inflammation. Allergy, 75, 1260–1263. 31713871 10.1111/all.14103

[jex2139-bib-0033] Muralidharan‐Chari, V. , Clancy, J. , Plou, C. , Romao, M. , Chavrier, P. , Raposo, G. , & D'Souza‐Schorey, C. (2009). ARF6‐regulated shedding of tumor cell‐derived plasma membrane microvesicles. Current Biology, 19, 1875–1885. 19896381 10.1016/j.cub.2009.09.059 PMC3150487

[jex2139-bib-0034] Nilsson, G. , Blom, T. , Kusche‐Gullberg, M. , Kjellén, L. , Butterfield, J. H. , Sundström, C. , Nilsson, K. , & Hellman, L. (1994). Phenotypic characterization of the human mast‐cell line HMC‐1. Scandinavian Journal of Immunology, 39, 489–498. 8191224 10.1111/j.1365-3083.1994.tb03404.x

[jex2139-bib-0035] Nolte‐’t Hoen, E. N. , van der Vlist, E. J. , de Boer‐Brouwer, M. , Arkesteijn, G. J. , Stoorvogel, W. , & Wauben, M. H. (2013). Dynamics of dendritic cell‐derived vesicles: High‐resolution flow cytometric analysis of extracellular vesicle quantity and quality. Journal of Leukocyte Biology, 93, 395–402. 23248328 10.1189/jlb.0911480

[jex2139-bib-0036] Obata, Y. , Toyoshima, S. , Wakamatsu, E. , Suzuki, S. , Ogawa, S. , Esumi, H. , & Abe, R. (2014). Oncogenic Kit signals on endolysosomes and endoplasmic reticulum are essential for neoplastic mast cell proliferation. Nature Communications, 5, 5715. 10.1038/ncomms6715 PMC4284665 25493654

[jex2139-bib-0037] Petersen, J. D. , Mekhedov, E. , Kaur, S. , Roberts, D. D. , & Zimmerberg, J. (2023). Endothelial cells release microvesicles that harbour multivesicular bodies and secrete exosomes. Journal of Extracellular Biology, 2, e79. 10.1002/jex2.79 PMC11080864 38939691

[jex2139-bib-0038] Pfeiffer, A. , Petersen, J. D. , Falduto, G. H. , Anderson, D. E. , Zimmerberg, J. , Metcalfe, D. D. , & Olivera, A. (2022). Selective immunocapture reveals neoplastic human mast cells secrete distinct microvesicle‐ and exosome‐like populations of KIT‐containing extracellular vesicles. Journal of Extracellular Vesicles, 11, e12272. 36239715 10.1002/jev2.12272 PMC9838129

[jex2139-bib-0039] Raposo, G. , Tenza, D. , Mecheri, S. , Peronet, R. , Bonnerot, C. , & Desaymard, C. (1997). Accumulation of major histocompatibility complex class II molecules in mast cell secretory granules and their release upon degranulation. Molecular Biology of the Cell, 8, 2631–2645. 9398681 10.1091/mbc.8.12.2631 PMC25733

[jex2139-bib-0040] Rialland, P. , Lankar, D. , Raposo, G. , Bonnerot, C. , & Hubert, P. (2006). BCR‐bound antigen is targeted to exosomes in human follicular lymphoma B‐cells. Biologie Cellulaire, 98, 491–501. 10.1042/BC20060027 16677129

[jex2139-bib-0041] Rueden, C. T. , Schindelin, J. , Hiner, M. C. , DeZonia, B. E. , Walter, A. E. , Arena, E. T. , & Eliceiri, K. W. (2017). ImageJ2: ImageJ for the next generation of scientific image data. BMC Bioinformatics, 18, 529. 29187165 10.1186/s12859-017-1934-z PMC5708080

[jex2139-bib-0042] Ryskalin, L. , Biagioni, F. , Lenzi, P. , Frati, A. , & Fornai, F. (2020). mTOR modulates intercellular signals for enlargement and infiltration in glioblastoma multiforme. Cancers, 12, 2486. 32887296 10.3390/cancers12092486 PMC7564864

[jex2139-bib-0043] Savina, A. , Furlán, M. , Vidal, M. , & Colombo, M. I. (2003). Exosome release is regulated by a calcium‐dependent mechanism in K562 cells. Journal of Biological Chemistry, 278, 20083–20090. 12639953 10.1074/jbc.M301642200

[jex2139-bib-0044] Schindelin, J. , Arganda‐Carreras, I. , Frise, E. , Kaynig, V. , Longair, M. , Pietzsch, T. , Preibisch, S. , Rueden, C. , Saalfeld, S. , Schmid, B. , Tinevez, J. Y. , White, D. J. , Hartenstein, V. , Eliceiri, K. , Tomancak, P. , & Cardona, A. (2012). Fiji: An open‐source platform for biological‐image analysis. Nature Methods, 9, 676–682. 22743772 10.1038/nmeth.2019 PMC3855844

[jex2139-bib-0045] Segura, E. , Nicco, C. , Lombard, B. , Véron, P. , Raposo, G. , Batteux, F. , Amigorena, S. , & Théry, C. (2005). ICAM‐1 on exosomes from mature dendritic cells is critical for efficient naive T‐cell priming. Blood, 106, 216–223. 15790784 10.1182/blood-2005-01-0220

[jex2139-bib-0046] Shelke, G. V. , Williamson, C. D. , Jarnik, M. , & Bonifacino, J. S. (2023). Inhibition of endolysosome fusion increases exosome secretion. Journal of Cell Biology, 222(6), e202209084. 37213076 10.1083/jcb.202209084 PMC10202829

[jex2139-bib-0047] Shelke, G. V. , Yin, Y. , Jang, S. C. , Lässer, C. , Wennmalm, S. , Hoffmann, H. J. , Li, L. , Gho, Y. S. , Nilsson, J. A. , & Lötvall, J. (2019). Endosomal signalling via exosome surface TGFβ‐1. Journal of Extracellular Vesicles, 8, 1650458. 31595182 10.1080/20013078.2019.1650458 PMC6764367

[jex2139-bib-0048] Skokos, D. , Le Panse, S. , Villa, I. , Rousselle, J. C. , Peronet, R. , David, B. , Namane, A. , & Mécheri, S. (2001). Mast cell‐dependent B and T lymphocyte activation is mediated by the secretion of immunologically active exosomes. Journal of Immunology, 166, 868–876. 10.4049/jimmunol.166.2.868 11145662

[jex2139-bib-0049] Smrž, D. , Bandara, G. , Zhang, S. , Mock, B. A. , Beaven, M. A. , Metcalfe, D. D. , & Gilfillan, A. M. (2013). A novel KIT‐deficient mouse mast cell model for the examination of human KIT‐mediated activation responses. Journal of Immunological Methods, 390, 52–62. 23357051 10.1016/j.jim.2013.01.008 PMC3602326

[jex2139-bib-0050] Smrž, D. , Kim, M. S. , Zhang, S. , Mock, B. A. , Smrzová, S. , DuBois, W. , Simakova, O. , Maric, I. , Wilson, T. M. , Metcalfe, D. D. , & Gilfillan, A. M. (2011). mTORC1 and mTORC2 differentially regulate homeostasis of neoplastic and non‐neoplastic human mast cells. Blood, 118, 6803–6813. 22053105 10.1182/blood-2011-06-359984 PMC3245204

[jex2139-bib-0051] Sundström, M. , Vliagoftis, H. , Karlberg, P. , Butterfield, J. H. , Nilsson, K. , Metcalfe, D. D. , & Nilsson, G. (2003). Functional and phenotypic studies of two variants of a human mast cell line with a distinct set of mutations in the c‐kit proto‐oncogene. Immunology, 108, 89–97. 12519307 10.1046/j.1365-2567.2003.01559.x PMC1782858

[jex2139-bib-0052] van der Vlist, E. J. , Arkesteijn, G. J. , van de Lest, C. H. , Stoorvogel, W. , Nolte‐’t Hoen, E. N. , & Wauben, M. H. (2012). CD4(+) T cell activation promotes the differential release of distinct populations of nanosized vesicles. Journal of Extracellular Vesicles, 1, 18364. 10.3402/jev.v1i0.18364 PMC3760647 24009884

[jex2139-bib-0053] Consortium, E.‐T. , Van Deun, J. , Mestdagh, P. , Agostinis, P. , Akay, Ö. , Anand, S. , Anckaert, J. , Martinez, Z. A. , Baetens, T. , Beghein, E. , Bertier, L. , Berx, G. , Boere, J. , Boukouris, S. , Bremer, M. , Buschmann, D. , Byrd, J. B. , Casert, C. , Cheng, L. , … Hendrix, A. (2017). EV‐TRACK: Transparent reporting and centralizing knowledge in extracellular vesicle research. Nature Methods, 14, 228–232. 28245209 10.1038/nmeth.4185

[jex2139-bib-0054] Verweij, F. J. , Bebelman, M. P. , Jimenez, C. R. , Garcia‐Vallejo, J. J. , Janssen, H. , Neefjes, J. , Knol, J. C. , de Goeij‐de Haas, R. , Piersma, S. R. , Baglio, S. R. , Verhage, M. , Middeldorp, J. M. , Zomer, A. , van Rheenen, J. , Coppolino, M. G. , Hurbain, I. , Raposo, G. , Smit, M. J. , Toonen, R. F. G. , … Pegtel, D. M (2018). Quantifying exosome secretion from single cells reveals a modulatory role for GPCR signaling. Journal of Cell Biology, 217, 1129–1142. 29339438 10.1083/jcb.201703206 PMC5839777

[jex2139-bib-0055] Wang, X. , Wu, R. , Zhai, P. , Liu, Z. , Xia, R. , Zhang, Z. , Qin, X. , Li, C. , Chen, W. , Li, J. , & Zhang, J. (2023). Hypoxia promotes EV secretion by impairing lysosomal homeostasis in HNSCC through negative regulation of ATP6V1A by HIF‐1α. Journal of Extracellular Vesicles, 12, e12310. 36748335 10.1002/jev2.12310 PMC9903130

[jex2139-bib-0056] Wu, B. , Liu, D. A. , Guan, L. , Myint, P. K. , Chin, L. , Dang, H. , Xu, Y. , Ren, J. , Li, T. , Yu, Z. , Jabban, S. , Mills, G. B. , Nukpezah, J. , Chen, Y. H. , Furth, E. E. , Gimotty, P. A. , Wells, R. G. , Weaver, V. M. , Radhakrishnan, R. , … Guo, W. (2023). Stiff matrix induces exosome secretion to promote tumour growth. Nature Cell Biology, 25, 415–424. 36797475 10.1038/s41556-023-01092-1 PMC10351222

[jex2139-bib-0057] Xia, Y. C. , Sun, S. , Kuek, L. E. , Lopata, A. L. , Hulett, M. D. , & Mackay, G. A. (2011). Human mast cell line‐1 (HMC‐1) cells transfected with FcεRIα are sensitive to IgE/antigen‐mediated stimulation demonstrating selectivity towards cytokine production. International Immunopharmacology, 11, 1002–1011. 21356342 10.1016/j.intimp.2011.02.017

[jex2139-bib-0058] Xiao, H. , Lässer, C. , Shelke, G. V. , Wang, J. , Rådinger, M. , Lunavat, T. R. , Malmhäll, C. , Lin, L. H. , Li, J. , Li, L. , & Lötvall, J. (2014). Mast cell exosomes promote lung adenocarcinoma cell proliferation—role of KIT‐stem cell factor signaling. Cell Communication and Signaling, 12, 64. 25311367 10.1186/s12964-014-0064-8 PMC4206705

[jex2139-bib-0059] Xu, M. , Ji, J. , Jin, D. , Wu, Y. , Wu, T. , Lin, R. , Zhu, S. , Jiang, F. , Ji, Y. , Bao, B. , Li, M. , Xu, W. , & Xiao, M. (2022). The biogenesis and secretion of exosomes and multivesicular bodies (MVBs): Intercellular shuttles and implications in human diseases. Genes & Diseases, 10(5), 1894–1907. 37492712 10.1016/j.gendis.2022.03.021 PMC10363595

[jex2139-bib-0060] Yin, Y. , Shelke, G. V. , Lässer, C. , Brismar, H. , & Lötvall, J. (2020). Extracellular vesicles from mast cells induce mesenchymal transition in airway epithelial cells. Respiratory Research, 21, 101. 32357878 10.1186/s12931-020-01346-8 PMC7193353

[jex2139-bib-0061] Zabeo, D. , Cvjetkovic, A. , Lässer, C. , Schorb, M. , Lötvall, J. , & Höög, J. L. (2017). Exosomes purified from a single cell type have diverse morphology. Journal of Extracellular Vesicles, 6, 1329476. 28717422 10.1080/20013078.2017.1329476 PMC5505001

