# Supplementary material for: Activation of the receptor KIT induces the secretion of exosome‐like small extracellular vesicles
Source: J Extracell Biol. 2024 Jan 23;3(1):e139. doi: 10.1002/jex2.139 (PMC11080788; doi:10.1002/jex2.139)
Supplement: Supplementary file 1 — Supporting Information [file JEX2-3-e139-s001.docx]

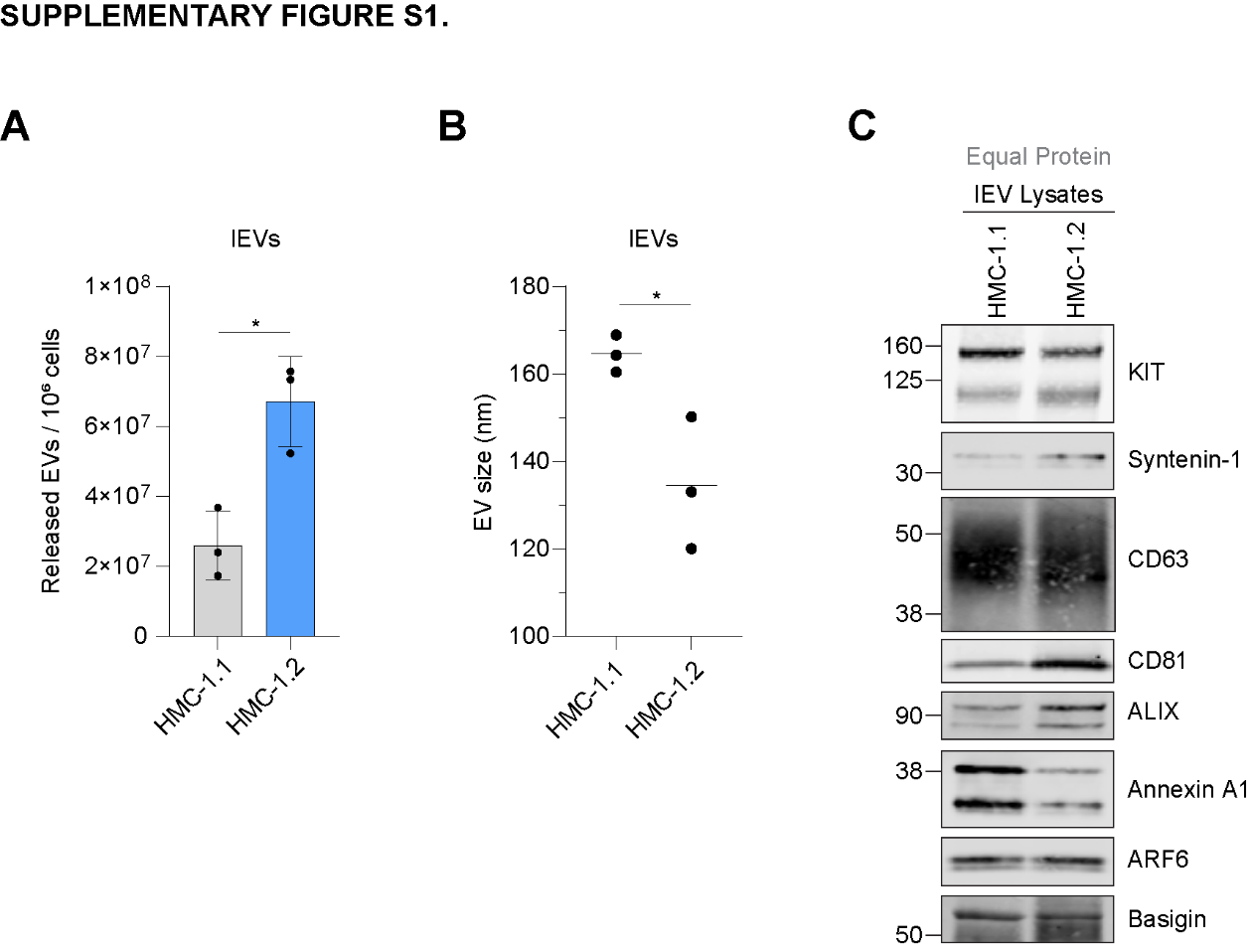


**Supplementary Figure S1. HMC-1.2 cells secrete more lEVs than HMC-1.1 cells.** (A) Quantitative comparison of the large EV (lEV) secretion from HMC-1.1 and HMC-1.2 cells. NTA-obtained data are represented as the mean ± S.D. from three independent experiments. (B) Size of lEVs secreted from HMC-1.1 and HMC-1.2 cells. The line represents the median from three independent experiments. (C) Immunoblot analysis of lEV lysates derived from HMC-1.1 or HMC-1.2 cells with the indicated antibodies. Equal EV protein (10 µg) was loaded. *, p<0.05; (unpaired t test).


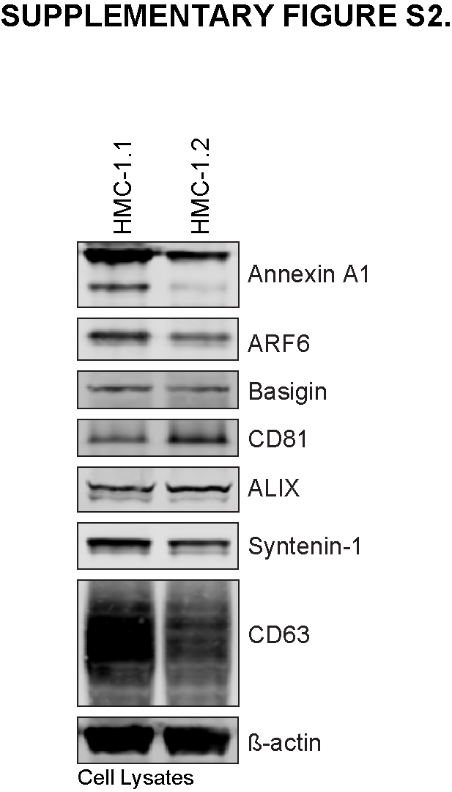


**Supplementary Figure S2. Cellular expression levels of selected proteins that are usually detected in EVs.** Lysates (30 µg of protein) of HMC-1.1 and HMC-1.2 cells were compared for protein expression levels by immunoblotting with the indicated antibodies.


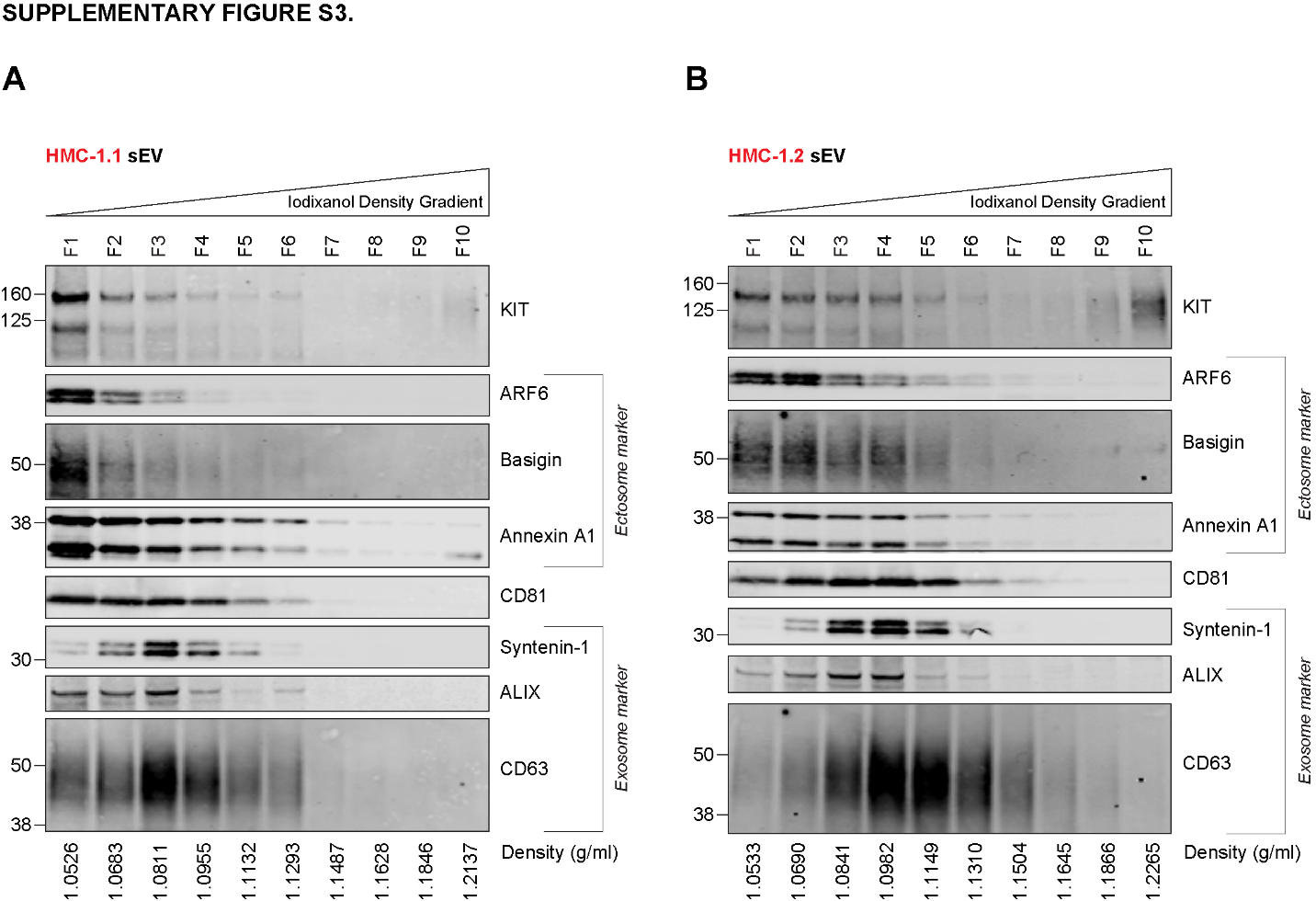


**Supplementary Figure S3. Analysis of sEVs derived from HMC-1.1 and HMC-1.2 cells by density gradients.** (A) sEVs isolated from the supernatant of 500x10^6^ HMC-1.1 cells were separated by bottom-loaded iodixanol density gradients. EVs floated into fractions 1-10 were analyzed by immunoblotting with the indicated antibodies. The density of each fraction was measured from blank gradients run in parallel and is indicated below the blots. (B) As in (A) but the gradient was performed with HMC-1.2-derived sEVs.

**SUPPLEMENTARY FIGURE S4.**


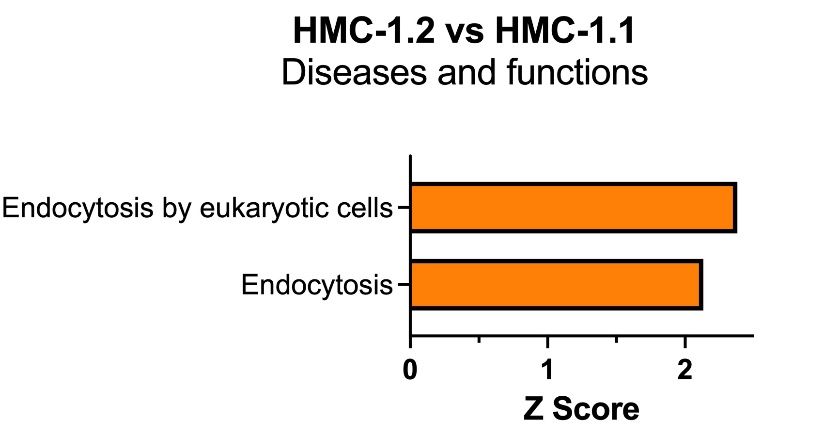


**Supplementary Figure S4. RNASeq analysis of HMC-1.1 and HMC-1.2 cells.** Z-Scores of Functions Annotations related to vesicular trafficking were predicted to be significantly increased in HMC-1.2 cells compared to HMC-1.1 cells. The analysis was performed using QIAGEN Ingenuity Pathway Analysis (IPA; QIAGEN Inc.; https://digitalinsights.qiagen.com/IPA) (Krämer *et al*, 2014). The overlap p values for the predicted functions were 3.2x10^-16^ for “endocytosis” and 1.6x10^-12^ for “endocytosis of eukaryotic cells”. The IPA analysis (p<0.05 and fold changes >1.3) was performed on RNASeq data from the following source (Bandara *et al*, 2023). The raw and normalized expression matrices from this data set are available in Gene Expression Omnibus under the accession ID GSE216446 (https://www.ncbi.nlm.nih.gov/geo/query/acc.cgi?acc=GSE216446).


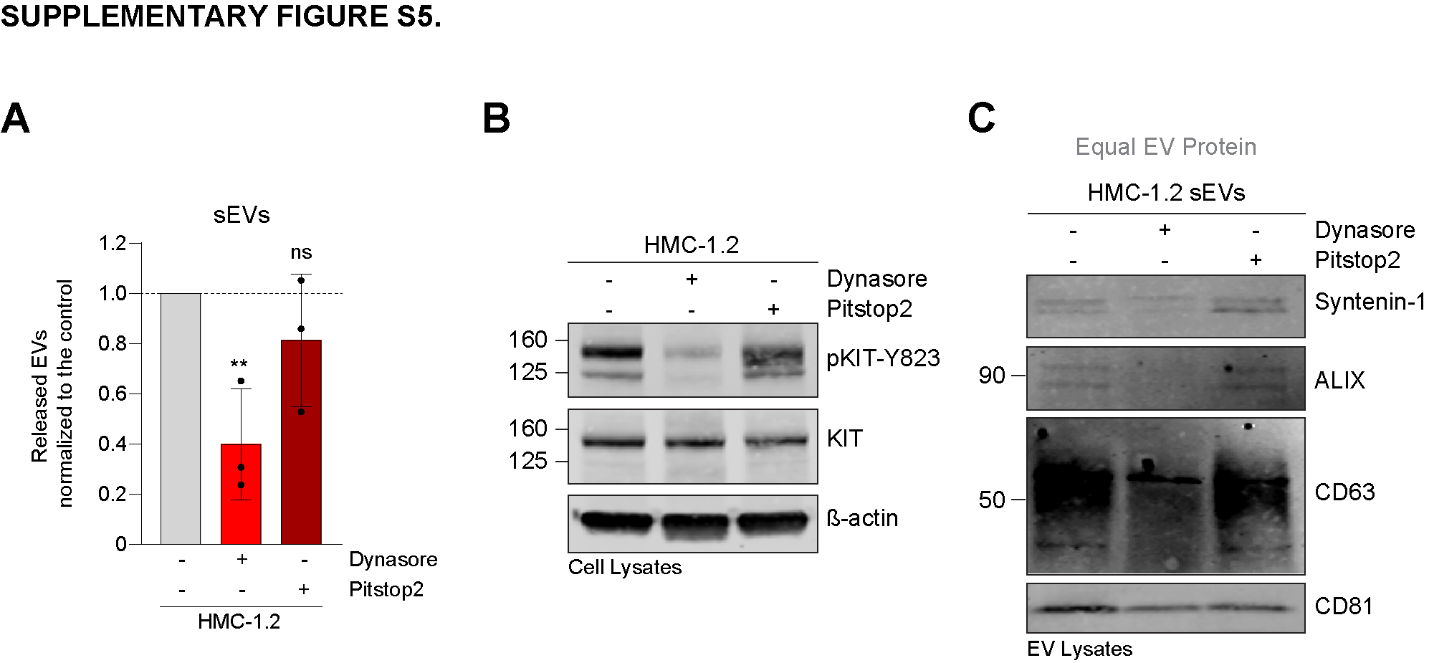


**Supplementary Figure S5. Inhibition of endocytosis reduces the secretion of sEVs by HMC-1.2 cells.** (A) Quantified sEVs secreted from HMC-1.2 cells incubated with the endocytosis inhibitors Dynasore and Pitstop2. (B) HMC-1.2 cell lysates (30 µg of protein) after incubation with Dynasore or Pitstop2 were analyzed by Western blotting with the indicated antibodies. (C) sEVs secreted from the cells in (B) were evaluated by immunoblotting. Equal EV protein (10 µg) was loaded. Ns, not significant; **, p<0.01; (unpaired t test).

**
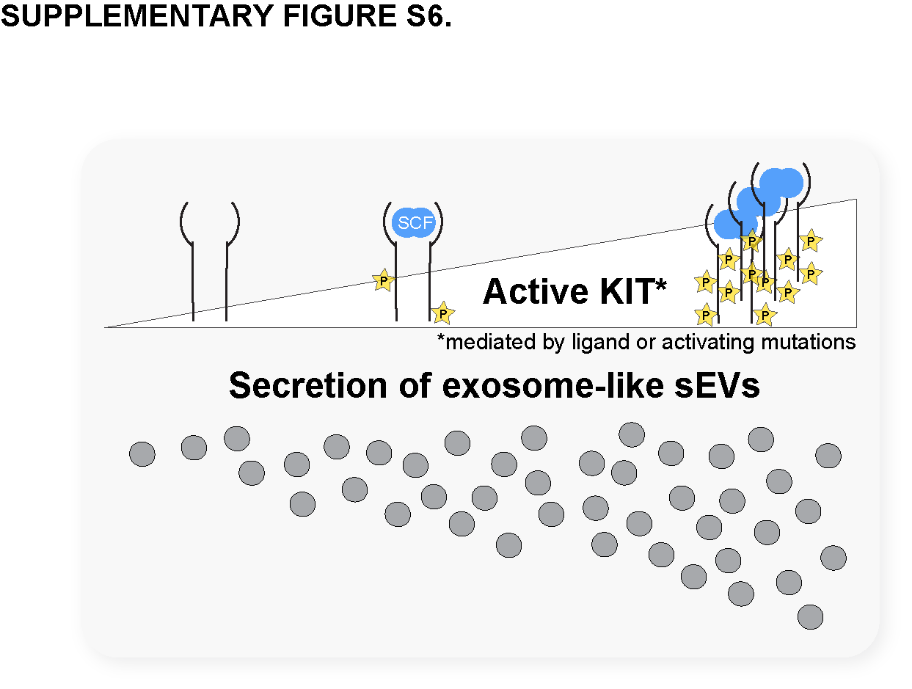
**

**Supplementary Figure S6. Model of the stimulated sEV secretion by KIT receptor activation.** The illustration shows that the KIT activity status, indicated by the gradient, influences the quantity of cell secreted exosome-like sEVs. The activity of KIT can be modified by its ligand stem cell factor (SCF) or by receptor activating mutations.


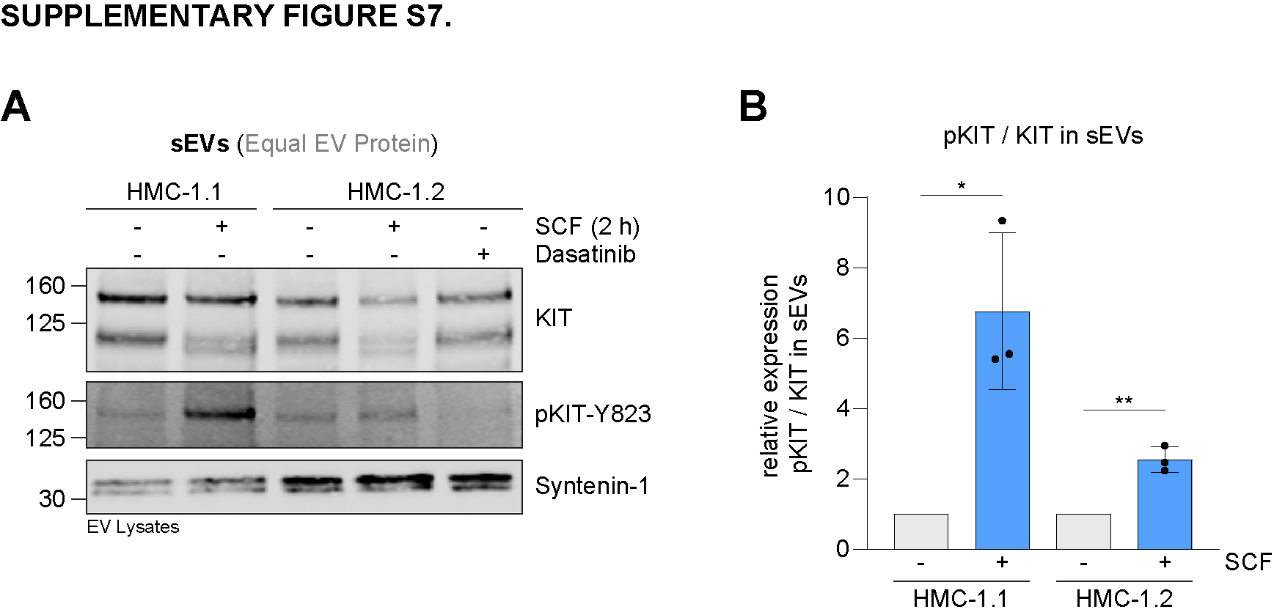


**Supplementary Figure S7. sEVs secreted from SCF-stimulated HMC-1.1 and HMC-1.2 cells contain increased phosphorylated KIT.** (A) sEVs released from unstimulated or SCF-stimulated HMC-1.1 or HMC-1.2 cells in the absence or presence of the KIT inhibitor dasatinib were analyzed by immunoblotting with the indicated antibodies. Equal EV protein (10 µg) was loaded. (B) Quantification of phosphorylated KIT (pKIT) expression in sEVs secreted from untreated or SCF-stimulated HMC-1.1 and HMC-1.2 cells (A). The relative expression of pKIT/KIT was normalized to sEVs derived from untreated cells in each cell line. Data are represented as the mean ± S.D. from three independent experiments. *, p<0.05; **, p<0.01 (unpaired t test).

**Additional References**

Bandara G, Falduto GH, Luker A, Bai Y, Pfeiffer A, Lack J, Metcalfe DD, Olivera A (2023) CRISPR/Cas9-engineering of HMC-1.2 cells renders a human mast cell line with a single D816V-KIT mutation: An improved preclinical model for research on mastocytosis. *Front Immunol* 14: 1078958

Krämer A, Green J, Pollard J, Jr., Tugendreich S (2014) Causal analysis approaches in Ingenuity Pathway Analysis. *Bioinformatics* 30: 523-530
